# Drivers of cervical cancer prevention and management in sub-Saharan Africa: a qualitative synthesis of mixed studies

**DOI:** 10.1186/s12961-023-01094-3

**Published:** 2024-02-08

**Authors:** Desta Debalkie Atnafu, Resham Khatri, Yibeltal Assefa

**Affiliations:** 1 Department of Health Systems Management and Health Economics, School of Public Health, Bahir Dar University, P.O.Box-79, Bahir Dar, Ethiopia; 2 School of Public Health, The University of Queensland, Brisbane, Australia; 3 International Centre for Evidence in Disability, London School Of Hygiene and Tropical Medicine, London, United Kingdom

**Keywords:** Cervical cancer, Screening programme implementation, Secondary prevention, Detractors, Enablers, Opportunities, Sub-Saharan Africa

## Abstract

**Background:**

Cervical cancer is a public health concern in the sub-Saharan Africa region. Cervical cancer screening is one of the strategies for detecting early precancerous lesions. However, many women have poor access to and utilization of screening services in the region. This review aimed to synthesize evidence on the challenges and opportunities of screening, early detection and  management of cervical cancer in sub-Saharan Africa.

**Methods:**

We conducted a structured narrative review of studies published in English. We included studies published from 1 January 2013 to mid-2022. Studies were selected following Preferred Reporting Items for Systematic reviews and Meta-Analyses (PRISMA) guidelines. Key search terms (detractors and enablers, cervical cancer screening, sub-Saharan Africa) were employed to identify studies from three electronic databases (HINARI, Science Direct, and PubMed). We also conducted searches on Google Scholar to identify relevant grey literatures. A thematic analysis was conducted and themes were identified, then explained using a socio-ecological framework (intrapersonal, interpersonal, organizational, community, policy levels).

**Results:**

We identified 60 studies in the final review. Cervical cancer screening and early detection and management programmes are influenced by drivers at multiple levels. Individual-level drivers included a lack of knowledge about cervical cancer and screening literacy, and a low risk in perception, attitude, susceptibility and perceived fear of test results, as well as sociodemographic characteristics of women. Interpersonal drivers were community embarrassment, women’s relationships with health workers, support and encouragement, the presence of peers or relatives to model preventive behaviour, and the mothers’ networks with others. At the organizational level, influencing factors were related to providers (cervical cancer screening practice, training, providers’ profession type, skill of counselling and sex, expert recommendation and work commitments). At the community level, drivers of cervical cancer screening included stigma, social–cultural norms, social networks and beliefs. System- and policy-level drivers were lack of nearby facilities and geographic remoteness, resource allocation and logistics management, cost of screening, promotion policy, ownership and management, lack of decentralized cancer policy and lack of friendly infrastructure.

**Conclusions:**

There were several drivers in the implementation of cervical cancer screening programmes at multiple levels. Prevention and management of cervical cancer programmes requires multilevel strategies to be implemented  across the individual level (users), community and organizational levels (providers and community users), and system and policy levels. The design and implementation of policies and programmes need to address the multilevel challenges.

**Supplementary Information:**

The online version contains supplementary material available at 10.1186/s12961-023-01094-3.

## Introduction

Cervical cancer (CC), caused by oncogenic types of human papillomavirus (HPV), is a well-known public health concern around the world, particularly in fragile health systems [1], and its precancerous stages entails several years before invasive cancer develops [2]. Having such a long latent period provides an excellent opportunity for early detection through effective screening and management [3].

Cervical cancer is the fourth most common preventable disease in women worldwide, with an estimated 569,847 cases per year [4]. Notably, cervical cancer accounted for 7.9% of all cancer cases in women worldwide [5]. Sub-Saharan Africa accounted for approximately 80% of the cancer cases identified in the late stages of the malignancy [6]. There was also 35 new cases per 100,000 women in a year [4].

Cervical cancer is also the leading cause of death in resource-constrained countries, accounting for 311,000 deaths worldwide annually [4]. Furthermore, unless a strong prevention strategy is implemented, the fatality rate for women is expected to rise by 42% that is 442,926 deaths in 2030 [8]. The mortality is currently much worse in low- and middle-income countries (LMICs), particularly in sub-Saharan Africa, where 85% of new cases and 87% of deaths have occurred owing to cervical cancer [9]. Sub-Saharan Africa is also a hotspot for cervical cancer ─ 35 new cases per 100,000 women and 23 deaths per 100,000 women occurring annually [4]. This disaster can be attributed to a lack of adequate cervical cancer information, access to existing screening services, stigma, late presence for diagnosis, and poor socio-cultural practices [7]. Absence of strong national cancer prevention policy and its weak implementation, as well as a lack of resources and technology to expand prevention activities, including screening services, is also exacerbating the problem [4, 9, 10].

Despite the availability of various screening modalities for resource-constrained settings and significant progress in increasing screening services globally, ensuring access to cervical cancer screening remains a challenge [11]. Cervical cancer screening and prevention programmes have received more attention in developed countries, whereas in fragile health systems, they have received less attention and consideration [12]. Globally, there are significant differences in the reduction of illnesses due to disparities in access to screening services. This is explained that, cervical cancer screening coverage is 63% in high-income countries and 19% in developing countries ─ with more than 40% gaps in screening coverage [11]. The estimated effective screening coverage in sub-Saharan Africa is 10%, while it is less than 1% in four West African countries and Ethiopia [13].

This low rate of prevention policy implementation, primarily screening coverage, followed by utilization, indicates that strategies for increasing accessibility of prevention modalities are not always in place for women in rural and hard-to-reach settings. Implementing cervical cancer screening in sub-Saharan Africa is a multifaceted issue with implications for individuals, interpersonal relationships, social, cultural, economic and organizational factors. Although several screening strategies for secondary prevention are available, expanding screening services is difficult and influenced by various individual, interpersonal, sociocultural, health system and contextual factors [14]. According to the literature, mothers’ acceptance of cervical cancer screening services was influenced not only by their own values and behavioural patterns but also by the quality of providers, community, sociocultural networks and organizations [15].

Member countries of the United Nations developed goals and strategies to ensure universal access to various services by 2030[16]. Importantly, the WHO agency for cancer research recommended expanding primary prevention through HPV prophylactic vaccination and secondary prevention through screening in the context of a well-resourced healthcare infrastructure [17]. Furthermore, the goals and recommendations emphasize equitable and sustainable access to all prevention services, including screening services for women aged 30 years and above [16].

Although early routine detection (screening) and treatment services can prevent up to 80% of precancerous lesions [18], many mothers in sub-Saharan Africa face barriers to accessing screening services [19]. Detractors who upset screening programme implementations may be linked to multiple socio-ecological factors [20]. These include a lack of health system structure, insufficient funding and high screening costs, provider attitudes towards routine screening services, a lack of understanding and advocacy for screening programmes, and a lack of health-seeking behaviour [19]. As a result, achieving cervical cancer prevention in resource-limited settings, such as sub-Saharan Africa, depends on identifying and implementing policies and schemes that can remove those situational drivers. However, given the region’s high cervical cancer burden, the political attention is insufficient [3, 21].

Despite WHO's aim to see 70 % coverage of high-performance screening tests, aspiring, 90% of women with pre-cancer would be treated, and 90% of women with invasive cancer would be managed throughout the world by 2030 [16]; however, African screening programme implementation practices are unfortunately low [17]. As a result, identifying the drivers which influence the implementation of screening programmes based on a socio-ecological framework is crucial. Therefore, this systematic narrative review aimed to assess the detractors and enablers of cervical cancer screening programme implementation practices to generate a summarized and reliable evidence that stakeholders in sub-Saharan Africa can pursue for a policy decision.

## Methods and materials

### Study design

We conducted a structured narrative review of the available evidence on cervical cancer screening, early detection and treatment in sub-Saharan Africa. The review protocol was registered using the PROSPERO International Prospective Register of Systematic Reviews and Meta-Analysis (ID = CRD42022303875).

### Literature search and selection

**Fig. 1:**
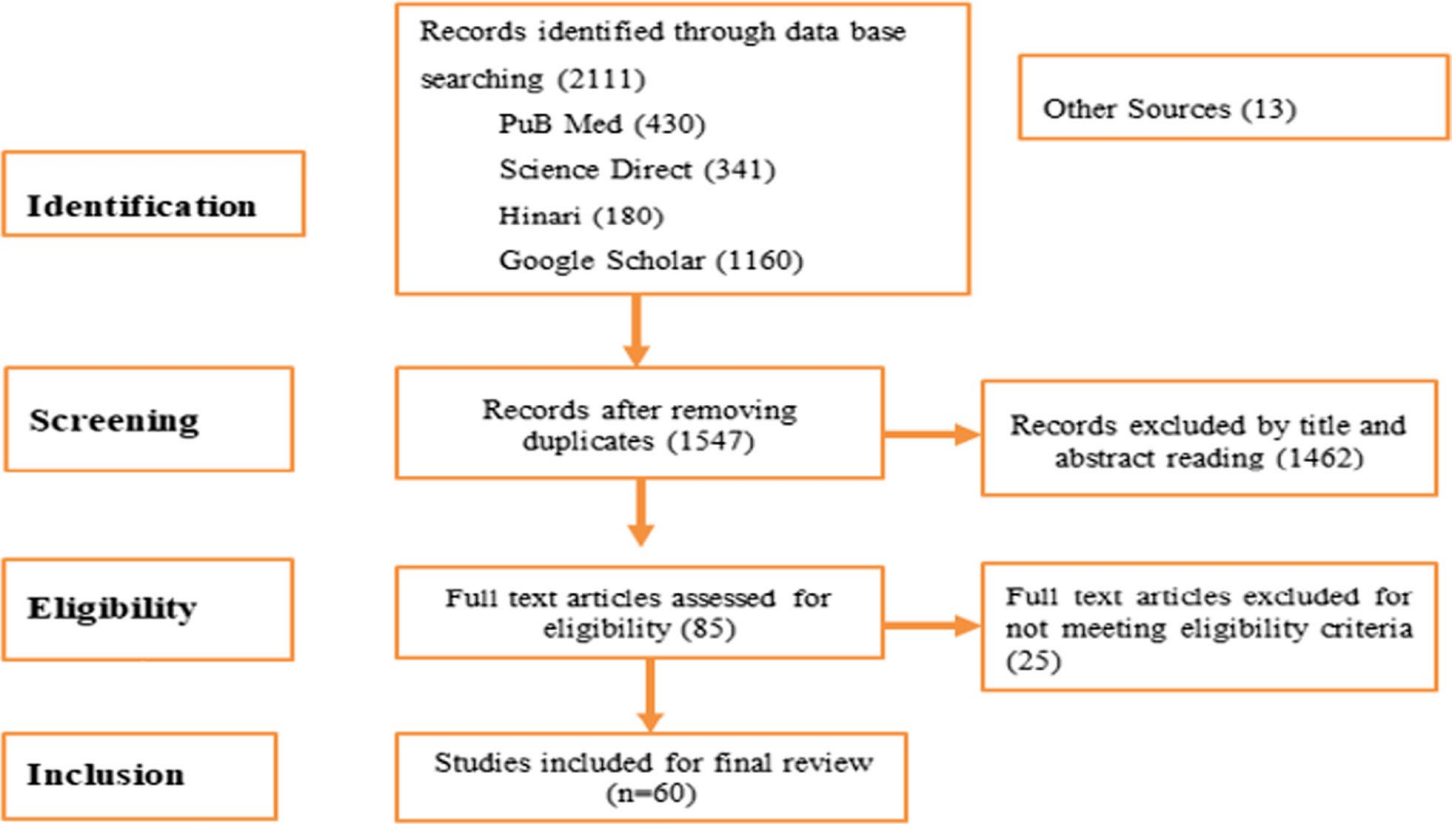
PRISMA flow chart for the study selection in the review of CC screening programme implementation 2022

The Problem or Population, Interventions, Comparison, Outcome, Context and Design(PICOCS) search strategies for electronic databases were utilized to identify relevant studies [22]. Medical subject headings (MeSH) and key terms relevant to the objective were also used in a literature search of the Science Direct, Hinari and PubMed databases. Google Scholar was also used to search for additional grey literature. The search strategy was built around three key phrases: detractors and enablers, cervical cancer screening programmes, and sub-Saharan African countries. The additional file presents the free text terms and medical subject headings (MeSH) used to search studies in the databases (Additional file 1). The search was restricted to studies published from 2013 onwards. We chose this time because critical recommendations for screen-and-treat strategies to prevent cervical cancer were updated this year, taking into account the context in which screen and treat will be implemented [16]. As a result, resource-limited countries began to establish and strengthen cervical cancer screening and treatment strategies in accordance with updated WHO guidelines [16]. The principal author (DDA) conducted the study selection to determine whether studies met the eligibility criteria using a three-stage Preferred Reporting Items for Systematic reviews and Meta-Analyses (PRISMA) guidelines: duplication removal, title and abstract selection, and full-text screening. After extensive discussions among the authors, studies were chosen by mutual agreement. Finally, the review included 60 studies (Fig. 1).

### Eligibility criteria

Participants, Interventions, Comparisons, Outcome, Context, and Study Design(PICOCS) methods was used to determine the eligibility criterion [22]. This review included healthcare providers; healthcare managers; women who had never intended, accepted or used CC screening; and women who had not had a hysterectomy. Women of any age were considered, although women aged 30–49 years were the prioritized as the target age for cervical cancer screening in low-resource settings [16, 23]. The intervention was the provision of low-cost CC screening services protocols for resource-limited settings (Visual Inspection With Acetic Acid (VIA), Visual Inspection with Lugol’s Iodine (VILI), Pap smear test) through self-swabbing at the community or facility level to prevent CC. Comparisons between women who intended/accepted/used the CC screening service and those who did not intend/accept/use it was assessed. This review included both literature with and without control groups. The outcomes were the drivers of the implementation of the CC screening programme. The context was sub-Saharan African countries (community and facility settings) where the studies were conducted. The review included studies with descriptive, observational (cohort, case–control, cross-sectional), qualitative and mixed-method designs. We also included all peer-reviewed studies published in English from 1 January 2013 to 30 June 2022. Commentaries, book sections, non-full articles, guidelines, generic, thesis, reports and systematic reviews were excluded.

### Quality appraisal

Three reviewers (DDA, RK and YA) independently and carefully assessed the risk of bias and methodological quality of the included studies until a discussion resolved disagreements on a consensus basis. The Agency for Healthcare Research and Quality (AHRQ) quality appraisal tool, which consists of nine-item questions about validity parameters, was used to assess the methodological and risk of bias of quantitative studies [24]. The methodological quality of the qualitative studies was evaluated using the CASP tool checklist [25]. An MMAT tool was used to assess the methodological quality of mixed-methods studies [26]. To determine the overall methodological quality, each included study’s percentage score was divided into three categories: 0–33% lower, 34–66% moderate and 67–100% high quality [27]. We did not exclude any study from this review based on the quality assessment results, believing that each study could contribute to our understanding of the various factors influencing cervical cancer screening programmes. The supplementary material includes detailed descriptions and explanations of quality assessments (Additional file 1).

### Data abstraction and synthesis

**Fig. 2:**
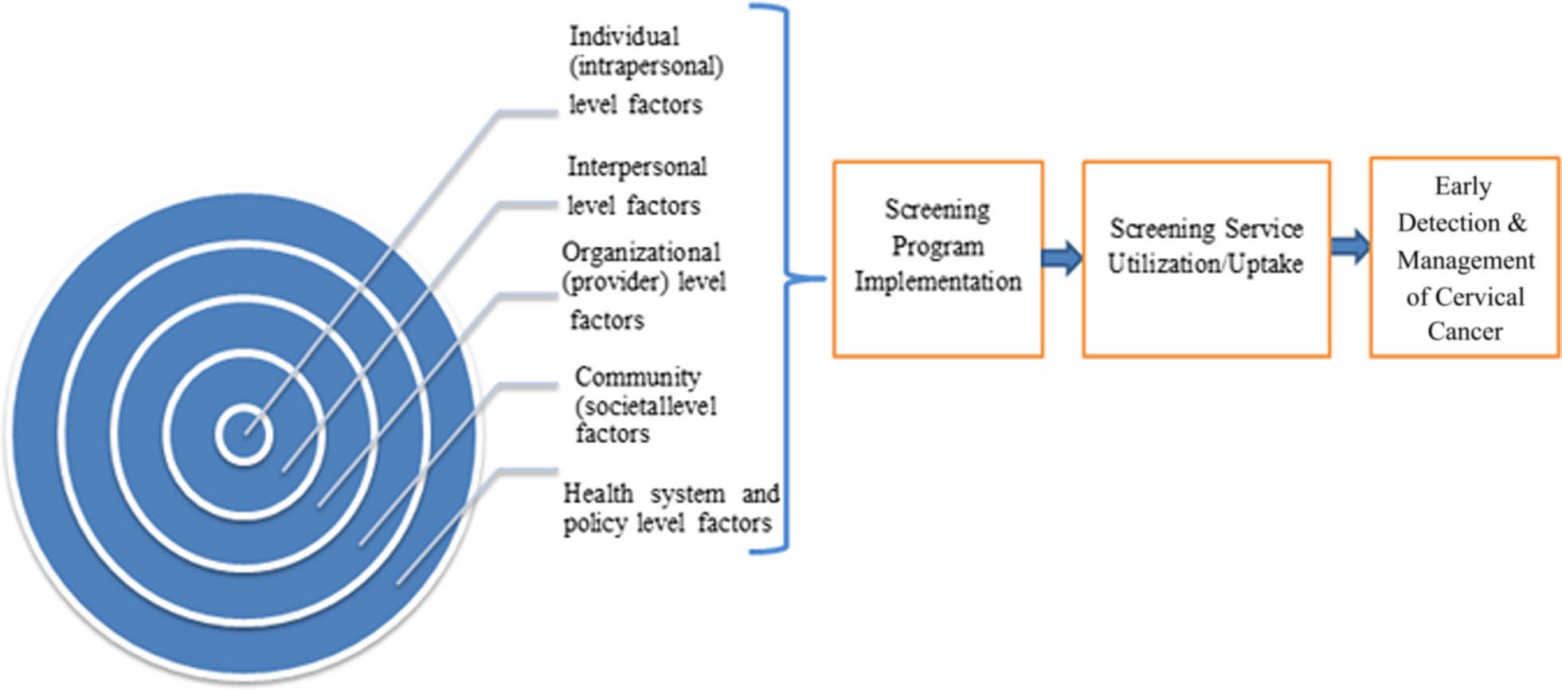
A socio-ecological framework of drivers and detractors of cervical cancer screening, early detection and treatment programmes in sub-Saharan Africa 2022

The principal author (DDA) abstracted data from included studies using a data abstraction template tailored to this systematic review. The second and third author (RK and YA) logically read and evaluated the first author’s data abstraction results. Disagreements between authors were thus resolved through a consensus-based discussion. As a result, the following key variables and characteristics were extracted: study information (author, year of publication, country of study, design), objective, methods (study population, sample size, non-response rate, sampling technique, data collection, analysis model), screening programme description (type of screening test, uptake, utilization, intention, accessibility, programme implementation), detractors and enablers, and key findings (Additional file 1). The findings of this review were narratively synthesized using a thematic synthesis framework to categorize detractors and enablers into main themes [28, 29]. The included studies were reviewed inductively to generate themes for cervical cancer screening detractors and enablers based on the domain of the social–ecological model (Additional file 1). The socio-ecological model (framework) was employed to organize and present [30] the analysis and synthesis of 60 full-text articles. Hence, as illustrated in the figure, this thematic synthesis identified five common themes that interact and influence cervical cancer screening, early detection and treatment programmes at various levels: individual, interpersonal, organizational (provider), community (sociocultural), health system and policy level factors (Fig. 2). A meta-analysis was not performed in this review due to the heterogeneity of the included studies in terms of study design, country and outcome measure.

## Results

### The search results

The review included a total of 60 studies. Figure 2 depicts the PRISMA flow chart of the studies chosen for this review.

### Characteristics and description of studies

The current review included 60 studies: 40 quantitative, 17 qualitative and three mixed-methods studies. Of the 40 quantitative studies, 39 studies were cross-sectional, whereas *n* = 17 were qualitative studies with a variety of qualitative designs, and the remaining (*n* = 3) were supplemented with mixed-method designs. Regarding the study outcome measure, 42 studies were conducted on the uptake of cervical cancer screening using either VIA/Pap smear tests, three on cervical cancer screening intention, five on cervical cancer screening awareness and the rest on screening acceptance, accessibility, norms and beliefs. Data were extracted from recent publications published between 2013 and 2022, with the majority of included articles (63%) published after 2018. The reviewed articles were piloted in 16 sub-Saharan African countries, among which the leading number of studies were conducted in Eastern Africa (55%t) and Western Africa (25%), primarily in Ethiopia (30%) and Ghana (11.7%). The study participants ranged in age from 10 to 74 years, even though the WHO recommends cervical cancer screening between the ages of 30 and 49 years. Data for quantitative studies were primarily collected through face-to-face interviews (66.7%), self-administered questionnaires (13.3%) and the rest of the studies did not put the techniques used. In contrast, in qualitative studies, the most common methods were focused group discussions (FGD) (71%) and key informant interviews (KII) (65%). The screening modalities in many of the studies (46.7%) were not clearly determined. Pap smear and VIA, on the other hand, were the screening modality used in 35.5% and 30% of the studies, respectively. In quantitative studies, the most common sampling strategies were multistage (28%) and systematic random sampling (26%), whereas in qualitative studies, purposive sampling (66.7%) was the most common.  The studies included in this review assessed for both detractors and enablers, and factors in 81% of quantitative studies were compared using regression analysis. In contrast, factors were assessed using content and thematic framework analysis modalities in 47% and 35% of qualitative studies, respectively. Only four studies drew on secondary data from demographic and health surveys. At the community level, approximately 33 studies were conducted, with 53 participants being women. Four of the women in the group were human immunodeficiency virus (HIV) positive. The characteristics of the study are included in Additional File 1.

### Detractors and enablers of the cervical cancer screening programme and cross-cutting themes

**Fig. 3:**
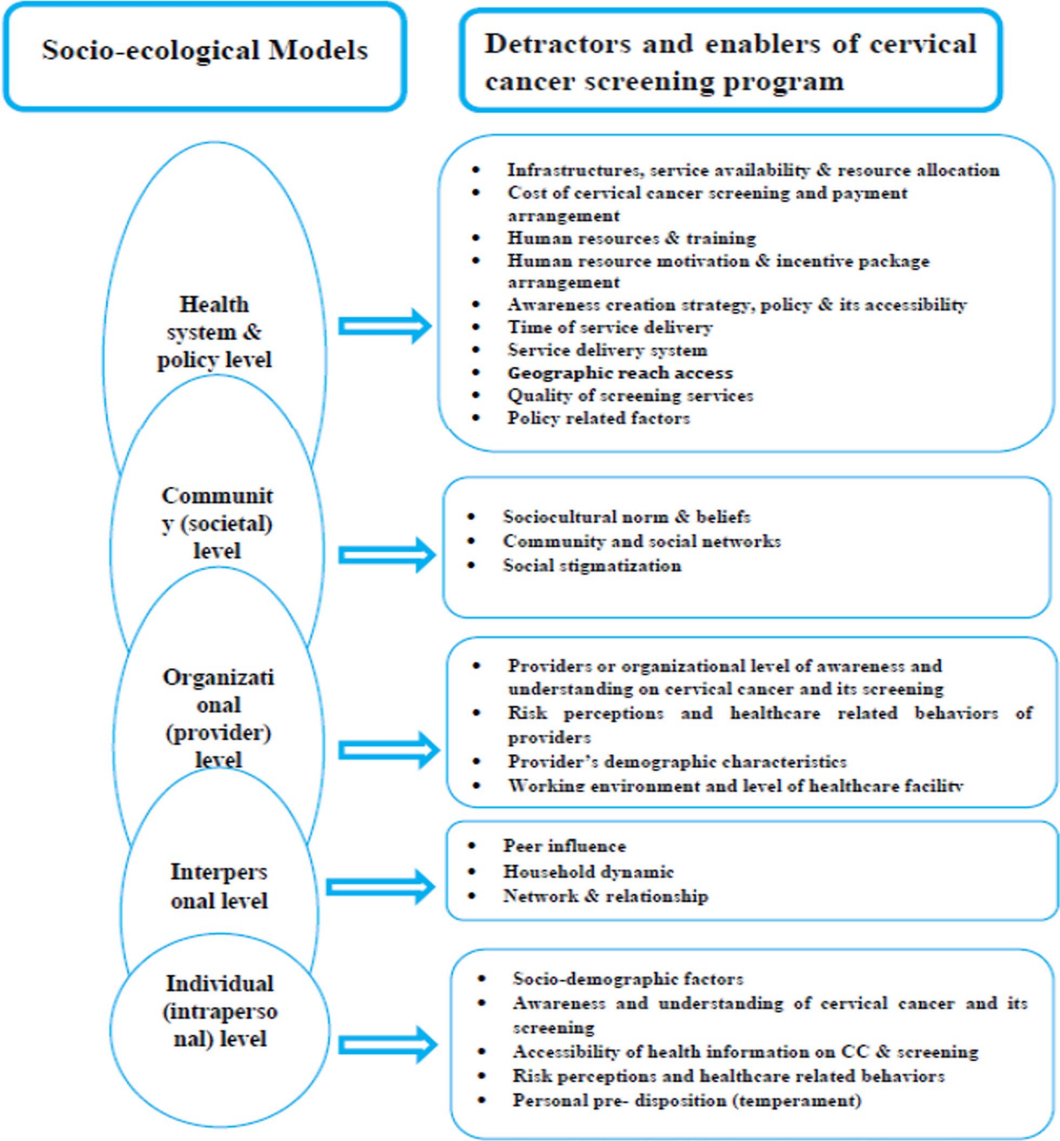
Detractors and enablers of cervical cancer screening programme implementation in sub-Saharan Africa

According to the review, the intent of stakeholders, detractors and enablers to function at individual, community, organizational or different levels of the healthcare system influenced cervical cancer screening programme implementation. Factors influencing implementation, uptake and intention to use cervical cancer screening programmes were assessed narratively in each theme to summarize the key results following the socio-ecological model. Figure 3 depicts the major barriers and enablers to women’s access to cervical cancer screening services in sub-Saharan Africa. The most common detractors reported by the reviewed articles were an embarrassment, the lack of a fiancé to pay for screening, poor knowledge of screening, partner refusal, provider attitude, sex, the cost of screening and the inaccessibility of services. Age, provider recommendation, financial incentives, family illness history, education status and other factors are common enablers. Table 1 organizes factors that detract from and motivate cervical cancer screening programmes based on the key findings from quantitative studies.

**Table 1:** Summary of the factors that influence cervical cancer screening programme implementation based on findings synthesized from quantitative studies, 2022

Factors	Correlation with screening		Statistically significant effect on screening		No. of studies	Conclusion
	Positive	Negative	Increasing	Decreasing		
Individual-level factors						
Age (≥ 30 years)	12	1	12	1	13	Facilitator
Educational status (≥ primary)	9	–	9		9	Facilitator
Income	7	–	8	3	10	Facilitator
Occupation (government	2	–	2	–	2	Facilitator
Employment (yes)	1	–	1	–	-	Facilitator
Residence (urban)	3	1	2	1	4	Facilitator
Religion (Christian)	1		1		1	Limited evidence
Knowledge (good)	13	2	13	1	26	Facilitator
Perceived susceptibility	8	2	8	1	10	Facilitator
Perceived fear ( test result, pain, removing womb	1	–	–	1	1	Limited evidence
Attitude (positive)	3	–	3	–	3	Facilitator
History of illness (family)	4	–	4	–	4	Facilitator
History of visiting health facility	1	–	1	–	–	Limited evidence
Interpersonal level factors						
Partners refusal (disapproval)	–	2	-	2	2	Barrier
Family size	1	–	1	–	1	Limited evidence
Peer attitude (positive subjective norm)	2	–	2	–	2	Facilitator
Organizational-level factors						
Provider recommendation	4	-	3	1	4	Facilitator
Provider knowledge	1	1	1	1	2	Limited evidence
Attitude of provider	1	2	1	2	3	Barriers
Source of information (provider)	2	–	2	-	2	Facilitator
Sex (male)	–	3	–	3	3	Barrier
Type of profession (physician)	–	1	–	1	1	Limited evidence
Facility working	1	1	1	1	2	Limited evidence
Community-level factors						
Social stigmatization	–	1	–	1	1	Limited evidence
Social values and beliefs	–	2	–	2	2	Barriers
Health system and policy-level factors						
Inaccessibility to healthcare services	–	4	–	4	4	Barrier
Cost of CC screening services	1		1	2	3	Barrier
Financial incentive (free treatment)	2	–	2	1	2	Facilitator
Health education programme	4	1	4	1	5	Facilitator
Long queues	–	1	–	1	1	Limited evidence
Long distance from healthcare facility	3	–	3	-	3	Barrier
Having health insurance coverage	4	–	4	–	4	Barrier
Satisfaction with screening services	1	1	1	1	1	Inconclusive
Quality of screening services	1	–	–	1	1	Limited evidence
Lack of national cancer prevention policies	–	1	-	1	1	Limited evidence
Healthcare involvement as bad	1	–	–	1	1	Limited evidence

### Intrapersonal level factors

We classified intrapersonal level of detractors and enablers into various subthemes such as awareness and understanding of cervical cancer and screening, risk perception and healthcare factors, attitude factors and sociodemographic characteristics. The uptakes of cervical cancer programs are influenced by knowledge and awareness of cervical cancer prevention. Twenty studies informed that low knowledge about CC and its screening among women decreases the early detection of cervical cancer [31–50]. Similarly, lack of access to information on CC [37] and screening services [35, 42, 45, 51–53], unawareness of where to be screened [54] and lack of health literacy [53, 55] were the potential detractors to uptake screening. On the contrary, high knowledge of cervical cancer can improve healthcare-seeking behaviours of people at risk of CC. Hence, being knowledgeable on methods of prevention [55–66], availability of cervical cancer screening services [51], the consequence of advanced cervical cancer [51] and the place of cervical screening services [37] has an input on the success of cervical cancer screening programme utilization.

Given the high burden of CC, studies reported that many women believed they were not at risk of cervical cancer and felt no need for Pap screening [34, 42, 44, 67]. On the other hand, few studies show that susceptibility or fear of getting cervical cancer influenced the preventive behaviours of women at risk. Therefore, perceived susceptibility was a dominant enabler for screening [31, 37, 42, 44, 57, 63, 68, 69]. Studies showed that screening has preventive health benefits for cancer, and the benefits of coming for screening outweigh the challenges accompanied by the screening process [65, 69]. In contrast, a study revealed a decreasing association between low perceived benefits and screening [55].

Furthermore, risk perception related detractors to screening initiation included free screening test results [36–38, 40, 45, 50, 51, 68, 70], worry about stigma [46], emotional costs in the form of the disease [48, 65], the pain of the gynecological examination and procedure [32, 38, 43, 47, 51, 54, 65, 71], fear of the unknown [48], and myths and misconceptions about removing of the womb [49]. In addition, embarrassment, namely feeling shy [32, 43, 71, 72], fear of invasion into their privacy [49], location of self-collection or privacy [46], and the assumption that sexual organs are private [48] were also found to be substantial barriers to screening.

In three of the reviewed studies, women’s intention to undergo cervical cancer screening was low due to negative attitudes and perceptions such as carelessness and negligence [36, 70], thinking screening unimportant [70] and being a virgin and hence not legible for screening [72]. On the contrary, women and influential people with direct and indirect attitudes towards cervical cancer screening were more likely to intend to be screened for cervical cancer [68, 73–75].

The WHO recommend that females between 30 and 49 years be screened for cervical cancer [16], although schedules vary by country. As a result, the age for screening was a concern for women, which determined utilization for CC screening. In the current review, 15 studies reported that age influences cervical cancer screening uptake. Except in two studies [59, 72], being older was a facilitator to screening uptake [36, 37, 46, 51, 56, 57, 60, 64, 75–79]. The educational status of women plays a paramount role in influencing cervical cancer prevention, and the fact that literate women have a better understanding of cervical cancer and its screening. In the current review, women whose educational level was primary and above had better awareness of cervical cancer screening, which increased the decision-making power and healthcare-seeking behaviour, and highly facilitated the utilization of the screening service [36, 62, 66, 76, 79–83].

In eight full-text articles included in this review, the economic condition of women has been discussed as a significant barrier and facilitator associated with screening status. Most women with medium to high monthly incomes were more likely to have screening services to various degrees. Women who earn ≥ 63 751Rwandan Franc (RWF) [7], have an annual household income of more than 30 000 Ethiopian Birr (ETB) [76], rich women [61, 83], perceived income of the household rich [63, 69], higher household wealth index [84] and average monthly income of greater than 1170 ETB [78] were the reported enablers in this review. In countries without universal health coverage, the screening programme cost was exhibited as a potential challenge. Individuals living in rural areas [52] and those with a lack of financial resources [38, 44, 45, 70, 84], low monthly income [37] and lack of money [40, 45, 55] stated the financial constraints as a barrier.

Personal predispositions (temperament) include a history of illness, perceived current health status, history of practicing early detection tests and taking therapy, history of contraceptive use, history of visiting health centres and history of substance use influenced cervical cancer screening initiatives. Being ill [71] and having signs and symptoms of cervical cancer [85] were associated with increased screening utilization. Similarly, women diagnosed as HIV positive [60, 80] and with a CD4 count of less than or equal to 200 cells/mm^3^ were significantly more likely to take up screening services than their counterparts. At the same time, those who did not use hormonal contraceptives [77] had a low utilization of pre-cancerous prevention services. Seven studies cited that the history of women or their family members’ for any disease was an important motive and facilitator for increasing cervical cancer screening programmes [26, 37, 64, 75, 79]. Similarly, this review showed that having no signs and symptoms [39, 54] and not getting sick or feeling healthy [40, 51, 72, 78] was the other explanation for the low cervical cancer screening performance. The odds of screening programmes was reduced among those who rated their healthcare involvement as moderate and below [82].

### Interpersonal level factors

A qualitative study showed that support and encouragement from the spouse was a known facilitator of cervical cancer screening [85]. However, in another quantitative study, husbands did not support and encourage women’s Pap smear screening [71] and this fact was registered as a likely barrier. Getting advice from friends and relatives [35] or persuasion by peers [43] was the main motivator of women to participate in cervical cancer screening programmes. The presence of peers or relatives to model preventive behaviour [43] enabled women to undergo screening since women could hear of the preventive programme package from a relative or friend compared with other sources.

In this review, we found that women with children were more likely to be screened for cervical cancer [80]. This was also similarly reported in the qualitative study [85]. The odds of screening utilization were significantly greater among women with a parity of more than five children [78]. At the same time, another study found that women with a parity of greater than seven deliveries were more likely to be screened [79]. Personal connections and networks with peers, neighbours or family members who had abnormal screening tests (cervical cancer diagnosis) are paramount to increasing knowledge about cervical cancer. Therefore, the tendency to have screening was increased. A study showed connections to those with model preventive behaviour were also a potential facilitating factor of cervical cancer screening [43]. Community embarrassment, such as a women’s relationship with health workers, was another deterrent to screening acceptance [46].

### Provider (organizational)-level factors

It was discovered that screening practices were correlated with training, profession and sex. Being cared for by male medical workers was a significant barrier to screening [32, 68], according to a study that found an association between female providers and screening practices [77]. [32, 68]. Similarly, the current research emphasizes that training was the primary driver of increased screening [86]. Having a doctor as the screening provider reduces the likelihood of cervical cancer screening uptake because the current review advised that CCS must be delivered by nurses and female providers [87].

CCS utilization was influenced by several provider-level factors. Among them, the ability to provide counselling, communications and run awareness campaigns or discussion forums were critical. According to the studies included in this review, screening programme implementation was hampered not only by low provider awareness [88] and knowledge [43] of cervical cancer and screening [88], but also by their inability to conduct patient-centred dialogue [47] and screening awareness creation campaigns [52]. Despite the findings of the reviewed studies, the odds of cervical cancer screening were four times higher among women whose primary source of information was health workers [61, 64]. Moreover, two studies found that the ability of good health professionals to counsel [56] and facilitate discussion forums with clients [51] increased the use of cervical cancer screening programmes. In contrast, only one study identified insufficient cervical cancer health education as a deterrent to screening [49].

Contextual factors such as low work commitment, providers’ negative attitudes towards clients and a negative attitude towards cervical screening were major detractors for the intention of having a screening test. As a result, four studies in this review identified healthcare providers’ attitudes as a major barrier to cervical cancer screening programmes [32, 47, 52, 88]. On the other hand, the encouragement and support for the screening test by health workers was a key motivator for women to follow their instructions. As a result, studies have shown that physician-initiated screening tests play a significant role in why women take screening tests [35, 65, 67, 75]. Four studies also discovered that women who had a health professional who did not recommend did not recommend screening were excluded from screening tests [34, 38, 40, 77].

Cervical cancer screening rates were lower among those who worked in cervical cancer screening centres [87]. This review, for example, discussed how the screening performance of health workers in a rural level III health centre was 70% lower than that of health workers in a level IV health centre [86]. Moreover, health workers who had worked in health centres that got organizational support for cervical cancer screening were more likely to screen for cervical cancer than staff from health centres without organizational support [86].

### Community and societal level factors

The community-level theme identified social–cultural norms, social networks, beliefs and religion as screening deterrents. Cultural detractors caused disparities in cervical screening uptake. The reviewed literature also vividly demonstrates the influence of sociocultural norms and beliefs regarding cervical cancer screening. As explored by a qualitative study, women who perceived and believed themselves to be healthy with no manifestation of gynaecological signs, took this as the absence of disease and stopped participating in cervical screening [41].

Another study found that social isolation and stigma discourage people from using cancer screening services [52]. Gender norms as a cultural construct emerged as the most significant barrier to cervical screening. Thus, cervical cancer linked to a women’s sexuality and reproductive organs contributes to the stigma and lowers the likelihood of screening [42]. Furthermore, most women were resistant to integrating screening into HIV care due to disease-specific stigma, even though it was a cost-effective and effective method of reaching out to unscreened women [48]. While integrating cancer literacy programmes into existing social networks may be an important source of social learning of screening behaviour, the review identified it as a cervical cancer screening enabler [42, 43].

Women’s religious beliefs and sociocultural values about cervical cancer also influence screening decisions. The negative beliefs of women about the Pap smear test, for example, embarrassing and painful [67], and religious and cultural practices [67, 68], were demotivating women from seeking screening tests. Some Christian churches, for example, did not believe in any disease screening test and did not allow women to undergo cervical cancer screening [48]. As a result, political intervention is required to overcome cultural detractors by developing culturally sensitive cervical cancer screening policies.

### Healthcare system and policy-level factors

Given the charter in South African Batho Pele principles and clients’ rights [38], which stated that women should have the right to receive needed healthcare within less than 5 km of healthcare facilities, women were having difficulty going for cervical cancer screening, primarily due to a lack of facilities [34, 71] and geographic remoteness [41, 47, 52, 53, 55, 60, 67], as reported in the reviewed studies. Although mobile clinics provide access to screening services for women who do not have alternative facilities or transportation to come into cities for screening, the lack of mobile clinics was also a potential barrier to attending screening services [38].

In this review, 11 articles reported that resource allocation and logistics management are significant barriers and public health concerns in screening processes. Furthermore, seven qualitative studies [41–43, 47, 50, 53, 55, 88] found that a lack of medical supplies and equipment was a common deterrent. Another impediment to screening implementation was a lack of screening and diagnostic tools, such as VIA [45]. Furthermore, stock out of supplies [49], was identified as a major challenge reported by the women studied.

Without a universal health coverage initiative, the direct and indirect costs of cervical cancer screening have been identified as a major barrier to programme implementation [52, 65]. As a result, this review proposed that, to increase screening uptake, cervical cancer screening services be provided free of charge, or a free payment arrangement for cervical cancer screening be established, similar to HIV/AIDS treatment services [32, 50, 85, 89]. Other reviewed articles also reported that the cost of screening procedures was unaffordable for women from rural areas, despite their willingness to screen [32, 42, 53, 60, 85]. As three reviewed studies suggest, expanding health insurance coverage was a likely enabler of the screening programme [60, 81, 84].

The human resource context was said to influence the implementation of the cervical cancer screening programme. Seven qualitative studies found that the most common barriers to cervical cancer screening uptake and implementation were human resource planning, management and skill level [41, 43, 45, 47, 55, 88, 90]. In Malawi, a lack of trained healthcare professionals was common in most cancer treatment facilities, linked to lower screening utilization [41]. The availability of limited health personnel in healthcare facilities was also cited as a barrier to accepting cervical cancer screening and treatment [90]. The ability of professionals to read smear results and their level of training [43, 45, 47, 55] were critical in influencing screening. The lack of a healthcare workforce makes programme implementation difficult, resulting in lower screening uptake. Similarly, provider and medical director turnover posed a unique challenge that hampered the quality of screening service delivery [88]. The availability of financial incentive packages for healthcare providers at the health facility, on the other hand, was emphasized to increase screening utilization [89].

Screening programme uptake has been known to be influenced by awareness creation, promotion strategies and media exposure. In this review, women’s understanding and trust in the screening delivery system were negatively impacted by poor sensitization and the absence of health education programmes on cervical cancer and its screening services [67]. In contrast, the use of text messages [89], radio campaigns [67] and a media brief on cervical cancer [35] aided in the implementation of the screening programme. Increased access to screening mass campaign information [36], health information [85] and media exposure [84] also contributed to the high availability of screening services.

The timing of screening service delivery practice and the scheduling of follow-up appointments were discovered to influence screening service uptake. The time constraints reported as a challenge of the screening programme in the four reviewed articles were long queues and overcrowded healthcare facilities [38, 49, 52, 65]. Furthermore, the inability to submit test results promptly or delay screening was a common reason for women to be hesitant to use a screening programme [47, 48]. Another barrier reported by one of the reviewed studies was an inconsistent appointment schedule and system [47].

The ownership and management system of the screening programme was discovered to have an impact on its implementation. A review article, for example, described a donor-driven screening system that was non-resilient, characterized by unintended donor interferences in the healthcare system, and resulted in uneven screening process implementation [41]. On the other hand, good partnerships and leverage among stockholders have been reported to promote screening utilization sustainability and equity in programme management [90].

Respondents in a reviewed study reported that environmental constraints in facilities, such as a lack of screening space and infrastructures used to protect women’s privacy during screening tests, influenced screening decisions [49, 88]. The novelty of the test [46] and the availability of high-quality screening services, on the other hand, increased women’s satisfaction [90]. Women’s satisfaction with the screening programme services influenced their use of screening tests, whereas poor satisfaction with screening services demotivated them from using additional screening services [82]. In this review, the most common detractors affecting women’s satisfaction that demotivate screening uptake were poor reception of CC screening facility [36], poor perceived quality of care [55, 77] and poor treatment effectiveness [47].

The involvement of medical personnel in the screening programme planning influenced the implementation of the screening service delivery process. The performance of a decentralization policy on the service delivery system was a critical enabler of the initiation of screening and treatment services [90]. Furthermore, the existing government’s political will and support are critical in expanding screening services. A study revealed in this review that political support in terms of financing the test, initiating change at the system level and making guidelines for a screening test procedure available had a growing effect on uptake [43, 86, 90]. A few studies investigated the role of national cancer policy in influencing screening services uptake. According to this review, the lack of a clear national policy and implementation guideline for cervical cancer prevention and control resulted in low screening service acceptance [42, 45, 55, 82]. In two qualitative studies, poor referral policies and the lack of a proper follow-up mechanism [49] were cited as detractors of screening service utilization by respondents [49, 55]. The identified health system gaps had a greater impact on the expansion of a screening programme [90]. An emerging innovative strategy, integrating cervical cancer screening with existing services such as Sectually Transmited Infection (STI), could potentially increase cervical cancer screening uptake [43, 85]. Changes made at the system level to allow for alternatives to the pelvic examination were identified as possible facilitators of cervical cancer screening utilization [43].

### Opportunities used to enhance the implementation of the CC screening programme

WHO established various frameworks, strategies and guiding principles in general, and in the health systems of sub-Saharan African countries in particular, to make accessibility of cervical cancer screening programme implementation easier for the most vulnerable groups of women. Some of the strategies and opportunities used by resource-limited countries to provide an efficient screening service programme at the grass roots level of the healthcare system, where most rural women benefited, were screening service integration with the existing reproductive healthcare and HIV/AIDS programme, provision of VIA screening services for free, expanding mobile clinics, initiation of universal health coverage, guideline settings and partnership development and utilization of existing social networks, health literacy campaign through media coverage, private sector involvement, and political willingness and leadership. Most African countries draft and initiate  the screening programme implementation guideline because developing cervical cancer prevention and control guidelines is useful in coordinating health workers through adequate information to have a successful cervical cancer screening and scale-up. Health workers who followed cervical cancer screening guidelines were more likely to screen than their counterparts [86].

The financial situation of women in sub-Saharan African countries is poor, which limits the use of screening programmes [2]. The inclusion of free screening service packages in those communities, on the other hand, is an important strategy used to promote preventive behaviour among women. Although free screening access was not promoted constantly, screening service utilization increased significantly following the free screening service policy [32, 50, 89]. Another theme that emerged from a qualitative data analysis that facilitated cervical cancer screening uptake was free cervical cancer treatment [85]. It is critical to strengthen partnerships between  stakeholders and key international organizations to increase capacity and resource allocation for screen-and-treat programs. In Tanzania, the public–private partnership in cervical cancer prevention was considered, and such leverage had a noticeable opportunity to increase screening and treatment services throughout the country [90]. The implementation of universal health coverage can potentially reduce direct medical expenditure for women of reproductive age, while at the same time, also improving their financial capacity to claim and access cervical cancer screening services. Women with health insurance were more likely than non-insured mothers to be screened for cervical cancer (AOR = 4.15; 95% CI: 1.52, 11.4) [60]. In addition, women with health insurance had a significantly higher proportion of Pap smear tests (60.3%, *p* ≤ 0.001) [81].

Similarly, women enrolled in an insurance system had a higher rate of cervical cancer screening practice (AOR = 2.05; 95% CI 1.7–2.5) [84]. Furthermore, the health systems of the countries under review had healthcare awareness campaigns to increase cervical cancer screening uptake. As a result, women who participated in the campaign had a much higher likelihood of using screening services [40]. Cervical cancer knowledge was a determinant factor for screening service utilization, and the rate of screening service uptake was higher among women from knowledgeable groups [56, 60].

Women were concerned about the gender of screening providers due to embarrassment [48, 49], and arranging a female-led approach to promoting and integrating cervical cancer care services with reproductive health services could play a key role in increasing the uptake of cervical cancer screening services. The gender of healthcare professionals who provide screening services to women determines the success of a screening programme. Mothers screened by female healthcare professionals were more likely to use screening services than those screened by males [68]. Furthermore, the influence of husbands and male peers was involved in increasing screening norms as male partners and peers attempted to guide women’s screening decision-making [43, 85, 89]. The allowance of cervical cancer screening services in private healthcare facilities and the establishment of mobile screening clinics was associated with the screening service programme. Attendance of women to private healthcare facilities for cervical cancer screening was a facilitator of screening uptake. The percentage of women screened for cervical cancer was nearly nine times greater in those of women who attended private health facilities (AOR = 8.9; 95% CI 2.8, 28.0) [56], whereas qualitative findings revealed that the expansion of mobile clinics was another means of accessing screening services for those women who did not have the means to travel into the city for screening [38].

Strong political will and leadership, which is an important policy-level initiative of the cervical cancer programme, not only resulted in changes at the healthcare system level that facilitate the cervical cancer screening programme, but also decentralized screening service delivery through the development of guidelines that a screening service can be administered [90]. The alignment of the screening services advocacy strategy in existing social networks and social norms improved screening service delivery implementation [30]. The dissemination of screening evidence within women’s social networks significantly influenced women’s level of knowledge about cervical cancer [42]. Similarly, expanding social learning of screening behaviour through existing social networks was a significant basis [43]. Ensuring an organized and opportunistic screening service strategy increased uptake and was an important component of cervical cancer screening prevention and control programmes. Screening services were integrated into existing health services in healthcare facilities to avoid missed opportunities for screening tests [42, 43].

Furthermore, an innovative approach of integrating screening tests into existing HIV care services facilitated cervical cancer screening [85]. Another factor that increased cervical cancer screening engagement was the use of peer networks to model screening behaviour. Similarly, another study found that social support is an important component of social networks, including marital relationships, when it was included as one of the factors facilitating cervical cancer screening programmes [67]. Family is a constituency in which the social support network has been profoundly shaped; hence, resilient social networks enable healthy behaviours such as cervical cancer screening utilization.

Mainstreaming media (radio and television stations) that reached the majority  of socioeconomic groups of the communities reduced disparities in healthcare information access, and women can be knowledgeable about cervical cancer screening [89]. As a result, long-term and large-scale broadcasting coverage about cervical cancer awareness greatly increased screening service use [85]. In addition, the proportion of women who had received cervical cancer education through the media was higher [67]. As a result, the odds of screening for cervical cancer were significantly higher in women who had received media education [35]. Similarly, a cervical cancer media literacy campaign resulted in a significantly higher proportion of screening tests [84].

## Discussion

This review identified several detractors and enablers of screening programmes in Sub Saharan Africa (SSA). The age of women has an impact on the adherence of cervical cancer screening, contradicted with earlier studies. Although young women had low preventative behaviour towards screening practices [68], being younger or older was a facilitator. An other study showed that women over 35 years had lower odds of using CC screening [59], contradicting the fact that the risk of acquiring cervical cancer increases with age. For this reason, women in such age  range decided to adhere to screening programmes. Thus, as WHO has strongly recommended cervical cancer screening tests, particularly for women aged 30+ years [92], programme owners and interested bodies in SSA should promote and make screening service packages available for all, regardless of age.

School-based education is pivotal for improving awareness of healthcare services. In this review, the formal education of women and their husbands was a key facilitator of screening programme implementation, consistent across the included articles. However, it was contradicted with a study conducted in Norway and Nepal where higher literacy did not make females more knowledgeable about the disease or increase attendance for cervical cancer screening, respectively [92, 93]. This indicates that formal education alone does not result in a behavioural change in the use of screening services. As a result, another strategy, such as education about cancer prevention and control using mainstream media in general and cervical cancer in particular is commendable. However, access to health education for promoting health, including screening, was a challenge in many resource-limited countries. According to the review, the intentions of women not to screen for cervical cancer may be attributed to a lack of awareness about cervical cancer and its screening in areas where school-based literacy is low and access to health information is limited. Our analysis also revealed that women’s knowledge of cervical cancer significantly impacted their decision to participate in screening programmes. Women who lacked knowledge and had a poor understanding of cervical cancer and VIA screening were less likely to be screened, supported by previous studies in which women’s unfamiliarity with cervical cancer and the benefit of screening tests were the most common barriers reported [94]. This indicates the importance of widely disseminating the healthcare message of screening programmes to the target audience. In addition, how and where eligible women obtain screening information should be carefully considered. Moreover, women who received screening information from relatives, peers, spouses or providers were more likely to be screened. This means that when expanding a screening programme, policy-makers must consider not only the opinions of relevant others, but also ensure a reliable source of information packages. In addition to support or encouragement from spouses, the presence of peers or relatives as a model of preventive behaviour was noted as a motivation for taking up screening [95], which was corroborated by our review findings. This could explain why, despite women being the primary decision-makers in screening, the use of societal links to model screening behaviour significantly influenced the willingness of mothers to enroll in the screening programme. Recognizing previous screening experiences for cervical cancer by peers and relatives was an important factor in increasing women’s willingness to accept screening. In other words, the presence of model preventive behaviour in the family increased knowledge of cervical cancer, removing fear and stigmatization of cervical cancer screening and treatment.

Previous researches revealed that living in a rural area contributed to women not being screened for cervical cancer [93, 96, 97], similar to some of the  studies in this review. Absence from screening programmes may be justified by a lack of education, long-distance travel, and differences in cultural and societal outlooks among rural communities. Low cervical cancer awareness and sensitization are common in rural areas, influenced by the loose integration between healthcare systems and local views and value systems.

Substantiating findings of previous studies [7, 19], our analysis found that low income and financial resources hindered Pap smear screening in 13.3% of the reviewed studies. The financial burden from unemployment might explain insufficient cervical cancer screening utilization. However, in a few of included studies, employed women were more likely to undergo screening given that they benefited from health insurance coverage.

Marriage or divorce was an underlying enabler for Pap screening tests, despite this result not holding true across the majority of  included studies. However, the implication of this factor is supported by our investigation that a partners’ refusal (disapproval) was a known barrier to screening uptake. Furthermore, several peer-reviewed articles revealed that husbands did not always support Pap smear screening; consistent with previous reports in which a lack of support from the husband was cited as a unique barrier to screening [93].

Corroborated by existing studies [93, 96–98], studies showed that women’s perception of cervical cancer and its prevention significantly influenced the uptake of screening services, with varying results across the reviewed studies. Studies explained that the practice of cervical cancer screening was low due to poor knowledge and perception, complemented by poor awareness of cervical cancer and screening [31]. Conversely, utilization of cervical cancer screening among women with a positive perception of their vulnerability to acquiring CC was greater than those with negative perceptions. It has been argued that practicing a healthy lifestyle can be improved as women’s tendency to self-susceptibility of a disease increases. Hence, undesirable attitudes of women, as the result of limited schooling and poor awareness about the benefit of screening, should be changed through increasing formal education and mass media communication. Similar to the current review, perceived seriousness of cervical cancer was a strong predictor of actual screening [67, 99] and perceived benefits of screening was also positively related to screening [100]. This implies awareness creation and knowledge-building interventions, which could reduce embarrassment, should be emphasized along with expanding cervical cancer screening programmes, to increase women’s awareness of cervical cancer as a fatal disease that can be prevented through screening.

Although the WHO has recommended that over 80% of women aged 35–59 years should undergo screening at least once during their lifetime, embarrassment or shyness to show private parts of body during pelvic examination and fear of test results [20, 23] were the main barriers for screening, similarly to a previous systematic review [97]. Therefore, psychological intervention should be implemented in SSA to improve women’s decision-making for undergoing screening. The attitude of women was considerably associated with cervical cancer screening uptake; however, results were inconsistent across the included studies. In some studies, positive attitudes have shown an improved involvement in screening uptake, while inconsistent results existed across studies. It has been debated that negative attitudes towards screening were influenced by culture and the belief that Pap smear tests were terrifying and hurting to spoil, requiring efforts to change cultural and behavioural detractors.

Provider gender had a role in cervical cancer prevention, and male screening providers were associated with low utilization, consistent with former studies that found higher utilization of screening among women due to having a female practitioner [101]. Culturally sensitive practices, such as males touching private parts (for example, cervix), may make women more embarrassed. Strengthening cervical cancer prevention efforts could involve community mobilization for better views and increasing female screening service providers managed by midwifery.

Healthcare providers’ psychotherapy, including counselling and support, increases women’s awareness of cervical cancer and leads to increased screening programme uptake [91]. Similarly, this review understood that the professionals’ ability to counsel and conduct discussion forums with clients were crucial for screening uptake. A study piloted in Ethiopia [102] also supported this findings that counselling about screening and skills to perform screening were connected with uptake of service. However, in Indonesia, a lack of health advocacy and skilled providers is a major deterrent [103]. Low uptake of screening services was not only the effect of women’s health illiteracy status but also a lack of suitable training for service providers [91]. As a result, policy-makers must take note of the availability of competent counsellors with good patient-centred communication skills and media for large-scale promotion. Furthermore, a continuous capacity building package on cervical cancer screening is suggested to improve staff attitudes and improve client-centred screening practices.

Despite staff attitudes reported as a common barrier to screening service expansions, healthcare workers’ recommendations and encouragement of women influenced screening service uptake. However, this finding was not consistently observed in this review, although there is a similar report elsewhere [104]. For this reason, trusted community affiliates and experts should encourage women to undergo screening, overcoming challenges and promoting strong decision-making on screening uptake.

The review found that stigma related to cervical cancer screening practices was the most common barrier, often linked to misconceptions – screening services might disclose HIV status. Similarly, the cost of CC treatment in women with confirmed cervical cancer were other reasons why women sometimes faced and divorce because of the fear of the cost of management, similar to previous studies [105, 107]. Women diagnosed with cervical cancer often face discrimination due to misconceptions about the screening and its connection to STIS  [105, 107]. This is because many wrong beliefs and taboos prevail in developing countries due to poor knowledge and promotional activities, exacerbating stigmatization. Programme owners should pledge policy changes and pilot interventions targeting sociocultural communities.

Cervical cancer screening and treatment readiness and accessibility were far from universal in resource-deficient countries [100]. Correspondingly, shortages of medical supplies, financial challenges and costs of already available screening services were identified to be the limiting barriers for taking up services, reported by a study conducted in LMICs [108]. Programme owners and policy-makers should expand service availability by either integrating with existing services or establishing new centres. Maintaining essential medical commodities associated with screening is also advisable. Screening care fee removal and the promotion of free screening services as well as insurance systems can also help escalate utilization in lower resource settings.

The absence of skilled experts for cervical cancer screening services delayed service delivery and decreases utilization [1, 11], corroborated by studies conducted elsewhere [109]. Lack of suitable training for service providers and attrition of trained personnel contributed to these drawbacks in preventing CC. However, skilled personnel availability in healthcare facilities did not guarantee screening uptake. In the presence of suitable health experts, for example, women might abstain from screening due to fear of positive results, greater backlog for screening, lengthy time to know test results and busy clinics [109], similarly reported in the current review. Therefore, the demand for cervical cancer prevention services by eligible women can be impeded by the healthcare experts performing tests and handling women.

Notwithstanding any guiding principles on cervical cancer screening programmes worldwide, cervical cancer screening programmes are not well practiced globally, particularly in sub-Saharan Africa[97]. Lack of national cancer prevention policies, frameworks and structure, and poor referral systems contributed to lower uptake (11, 20). It can also be concluded that the introduction and implementation of cervical cancer prevention and control programmes in the healthcare systems of the studied countries were in their infancy. Sector organizations such as WHO should streamline policy and strategic ambitions, while increasing political determination on screening policy structures to improve cervical cancer screening practice, early detection and management.

### Limitation of the review

This review is not without limitations. A larger number of studies were found from Eastern and Western countries of sub-Saharan Africa, mainly Ethiopia and Ghana. The reflections from Ethiopia and Ghana might overlook the included studies in the review and thus, the conclusions might be impractical for other scenarios and areas. Some of the electronic databases were not yet accessed, and pertinent studies might be missed because of lack of access to the databases (lack of subscription). The heterogeneity of the reviewed studies due to differences in outcome measures and study settings is one of the problems that we could not come across to show the pooled effect of the cervical cancer screening programme implementation using a meta-analysis and thus, estimating the pooled effect size is difficult. This review did not consider studies on the self-sample collection HPV testing method, despite its importance in overcoming barriers faced by women in resource-limited settings where embarrassment and shortage of sample takers is a problem. Thus, we urge further future studies to complement the limitation.

### Policy implication of the study

Policy decision-makers and relevant collaborators having stakes in expanding the prevention and control of cervical cancer using early screening of women at risk might able to obtain evidences from the results of this review, with consideration of countries programme context. Screening service mainstreaming and integration in existed healthcare services might also improve screening availability and utilization. Moreover, promotional activities such as awareness and information campaigns through mass media could also induce more uptakes. Peer learning programmes using model behaviours among women with whom they trusted might be one strategy to sensitize eligible members about the programme. This review also identified the existence of inequity in screening service delivery, where the rural and those aged were systematically unreachable by the screening programme. Often, the screening programme was not pro-poor, and those wanted to be screened feared a catastrophic expenditure, and only those who can afford the expense decided to be screened. Thus, policy-makers should close this inequity by using an exemption policy for screening charge, like other essential healthcare services, despite the exemption of screening services might lead to efficiency. Screening quality and referral systems that are managed within the health system can be regulated to augment screening service utilization. This synthesis also has a policy implications where screening programme planners should scale-up essential services and make available critical supply and medicines. The review advised to have a respectful, compassionate and careful screening services, in which embarrassment is an obsolete problem associated with unfriendly behaviour of care providers. Institutional and regulatory tools must be in place to make an authentic and systemic change in service accessibility. Furthermore, stigma, misconceptions about CC screening test and fear of test results still persisted, demanding effective awareness campaigns targeting those in rural and uneducated settings. Couples education might also be imperative as the review distinguished divorce as a known barrier for screening.

## Conclusions

Screening is an effective prevention strategy for cervical cancer, a serious public health danger in sub-Saharan Africa. However, this review identified detractors and enablers to screening programme implementation. Consequently, the application of secondary prevention strategies for early detection, management and reduction of cervical cancer in the region has been hindered by feasibility and infrastructure-related drivers. Thus, due emphasis should be given while designing, implementing and monitoring the programmes to increase utilization of screening services for the early detection of cases and management. Cervical cancer screening programmes in the region must be expanded and harmonized at various levels, targeting not only at the individual level, but also at healthcare providers, community, and health system and policy levels. Cervical cancer prevention services should be well organized and consider the mass advocacy of the services, adequate financial resources, infrastructure and trained human resources. Moreover, upcoming studies should also be conducted on the implication of contextual factors on programme accessibility and use, including cultural, political and economical factors.

### Supplementary Information

**Additional file 1.** **Supporting information 1:** Search strategies; **Supporting information 2:** Quality appraisal of the included studies; **Supporting information 3:** Characteristics and key findings of the included studies.

## Data Availability

All relevant materials and data supporting the findings of this study are freely available. All relevant data are within the manuscript and its Supporting Information files. When necessary, please contact **destad2a@gmail.com**.
