# Supplementary material for: Drivers of cervical cancer prevention and management in sub-Saharan Africa: a qualitative synthesis of mixed studies
Source: Health Res Policy Syst. 2024 Feb 8;22:21. doi: 10.1186/s12961-023-01094-3 (PMC10851545; doi:10.1186/s12961-023-01094-3)
Supplement: Supplementary file 1 — Additional file 1. Supporting information 1: Search strategies; Supporting information 2: Quality appraisal of the included studies; Supporting information 3: Characteristics and key findings of the included studies. [file 12961_2023_1094_MOESM1_ESM.docx]

**Additional Materials**

**Additional information 1: Search strategies**

1. **PubMed search strategy**

|  | (‘Barriers’ OR ‘Sociocultural barriers’ OR ‘Culture’ OR ‘Challenges’ OR ‘Limitation’ OR ‘Gaps’ OR ‘Weakness’ OR ‘Failure’ OR ‘Cost’ OR ‘Deterring factors’) AND (‘Enablers’ OR ‘Education’ OR ‘Facilitators’ OR ‘Opportunities’ OR ‘Attitude’ OR ‘Strength’ OR ‘Enabling factors’ OR ‘Success’ OR ‘Cost effectiveness’) AND (‘Program’ OR ‘System’ OR ‘Programme’ OR ‘Scale-up’) AND (‘Implementation’ OR ‘Delivery’ OR ‘Performance’ OR ‘Efficiency’ OR ‘Monitoring’ OR ‘Evaluation’) AND (‘Cervical cancer’ OR ‘Cancer of Cervix’ OR ‘human papilloma viruses’ OR ‘HPV’ OR ‘Uterine cervical neoplasms’ OR ‘Uterine’ OR ‘Cervical’ OR ‘Neoplasms’ OR ‘Cancer’) AND (‘Diagnosis’ OR ‘Screening’ OR ‘Mass screening’ OR ‘Early detection of cancer’ OR ‘Detection’ OR ‘Cervical cancer control’ OR ‘Cervical cancer prevention’ OR ‘Prevention’ OR ‘Secondary prevention’ OR ‘Cervical cancer secondary prevention’ OR ‘HPV screening’ OR ‘Pap smear’ OR ‘HPV DNA test’ OR ‘Self-sampling’ OR ‘Colposcopy’ OR ‘Cryotherapy’ OR ‘LEEP’ OR ‘Papanicolaou test’ OR ‘Pap test’ OR ‘VIA’ OR ‘Visual inspection with acetic acid’) AND (‘Uptake’ OR ‘Utilization’) |
| --- | --- |
|  | (“Barriers” OR “Challenges”) AND (“Facilitators” OR “Opportunities”) AND (“Cervical cancer”) AND (“Screening”) AND (“Utilization”) |
|  | (‘Angola’ OR ‘Benin’ OR ‘Botswana’OR ‘Burkina Faso’ OR ‘Upper Volta’ OR ‘Burundi’ OR ‘Cabo Verde’ OR ‘Cameroon’ OR ‘Central African Republic’ OR ‘Chad’ OR ‘Comoros’ OR ‘Congo’ OR ‘Zaire’ OR ‘Congo’, ‘The Democratic Republic’) AND (‘Cote dIvoire’ OR ‘Ivory Coast’) AND ‘Djibouti’ OR ‘Equatorial Guinea’ OR ‘Eritrea’OR ‘Ethiopia’ AND (‘Gabon’ OR ‘Gabonese’) AND (‘Gambia’ OR ‘Ghana’ OR ‘Guinea’ OR ‘Guinea-Bissau’ OR ‘Kenya’ OR ‘Lesotho’ OR ‘Liberia’ OR ‘Libya’ OR ‘Madagascar’ OR ‘Malawi’ OR ‘Mali’ OR ‘Mauritania’ OR ‘Mauritius’ OR ‘Morocco’ OR ‘Mozambique’ OR ‘Namibia’ OR ‘Niger’ OR ‘Nigeria’ OR ‘Papua New Guinea’ OR ‘Rwanda’ OR ‘Sao Tome and Principe’ OR ‘Senegal’ OR ‘Sierra Leone’ OR ‘Somalia’ OR ‘South Africa’ OR ‘St Vincent’ and ‘The Grenadines’ OR ‘Sudan’ OR ‘Swaziland’ OR ‘Zanzibar Syria’ OR ‘Tanzania’ OR ‘Togo’ OR ‘Uganda’ OR ‘Zambia’ OR ‘Zimbabwe’) |
|  | ‘Sub Saharan Africa’ [mesh] |
|  | #1 AND #2 |
|  | #1 AND #2 AND #3 AND #4 AND #5 |

1. **Hinari search strategy**

|  | Barriers, facilitators, and opportunities in the implementation of cervical cancer screening program in sub-Saharan Africa |
| --- | --- |

1. **Science Direct**

|  | ((‘Barriers’ OR ‘Challenges’) AND (‘Facilitators’ OR ‘Opportunities’) AND (‘Cervical cancer’) AND (‘Screening’) AND (‘Utilization’) AND (‘Sub Saharan Africa’)) |
| --- | --- |

1. **Google Scholar**

|  | (("Barriers") AND ("Facilitators") AND ("Cervical cancer") AND ("Screening") AND ("Sub Saharan Africa")) |
| --- | --- |

**Additional information 2: Quality appraisal of the included studies**

1. Supplementary material: Quality appraisal of the included quantitative studies (cross-sectional) on cervical cancer screening program implementation.

| Author’s last name, Publication year | Was the sample representative of the target population? | Were study participants recruited in an appropriate way? | Was the sample size adequate? | Were the study subjects and the setting described in detail? | Was the data analysis conducted with sufficient coverage of the identified sample? | Were objective, standard criteria used for the measurement of the condition? | Was the condition measured reliably? | Was there appropriate statistical analysis? | Are all important confounding factors/subgroups/differences identified and accounted for? | Were subpopulations identified using objective criteria? | Overall quality |
| --- | --- | --- | --- | --- | --- | --- | --- | --- | --- | --- | --- |
| 1.Abiodun OA. et al, 2014 ([31](file:///C:\Users\user\Desktop\CC%20Review-sept%203,%202022_RKinputs%20-%20(Autosaved).docx#_ENREF_94)) | Yes | Yes | Yes | No | Yes | Yes | Yes | Yes | Yes | Yes |  |
| 2.Téguété I. et al, 2021 ([89](file:///C:\Users\user\Desktop\CC%20Review-sept%203,%202022_RKinputs%20-%20(Autosaved).docx#_ENREF_94)) | Yes | Yes | Yes | Yes | No | Yes | Yes | No | No | Yes |  |
| 3. Belay Y. et al, 2020 ([56](file:///C:\Users\user\Desktop\CC%20Review-sept%203,%202022_RKinputs%20-%20(Autosaved).docx#_ENREF_94)) | Yes | Yes | Yes | Yes | Yes | Yes | Yes | Yes | Yes | Yes |  |
| 4.Hallidu M. et al, 2021 ([32](file:///C:\Users\user\Desktop\CC%20Review-sept%203,%202022_RKinputs%20-%20(Autosaved).docx#_ENREF_94)) | Yes | Yes | Yes | Yes | Yes | Yes | Yes | Yes | Yes | Yes |  |
| 5.Nega AD. at al, 2018 ([80](file:///C:\Users\user\Desktop\CC%20Review-sept%203,%202022_RKinputs%20-%20(Autosaved).docx#_ENREF_94)) | Yes | Yes | Yes | Yes | Yes | Yes | Yes | Yes | Yes | Yes |  |
| 6. Erku DA. et al, 2017 ([57](file:///C:\Users\user\Desktop\CC%20Review-sept%203,%202022_RKinputs%20-%20(Autosaved).docx#_ENREF_94)) | Yes | Yes | Yes | Yes | Yes | Yes | Yes | Yes | Yes | Yes |  |
| 7. Ndateba I. et al, 2021 ([58](file:///C:\Users\user\Desktop\CC%20Review-sept%203,%202022_RKinputs%20-%20(Autosaved).docx#_ENREF_94)) | Yes | Yes | Yes | Yes | Yes | Yes | Yes | Yes | Yes | Yes |  |
| 8. Kasim J. et al, 2020 ([66](file:///C:\Users\user\Desktop\CC%20Review-sept%203,%202022_RKinputs%20-%20(Autosaved).docx#_ENREF_94)) | Yes | Yes | Yes | Yes | Yes | Yes | Yes | Yes | Yes | Yes |  |
| 9.Emru K. et al, 2021 ([33](file:///C:\Users\user\Desktop\CC%20Review-sept%203,%202022_RKinputs%20-%20(Autosaved).docx#_ENREF_94)) | Yes | Yes | Yes | Yes | Yes | Yes | Yes | Yes | Yes | Yes |  |
| 10. Natae SF. et al, 2021 ([51](file:///C:\Users\user\Desktop\CC%20Review-sept%203,%202022_RKinputs%20-%20(Autosaved).docx#_ENREF_94)) | Yes | Yes | Yes | Yes | Yes | Yes | Yes | Yes | Yes | Yes |  |
| 11. Kimondo FC. et al, 2021 ([54](file:///C:\Users\user\Desktop\CC%20Review-sept%203,%202022_RKinputs%20-%20(Autosaved).docx#_ENREF_94)) | Yes | Yes | Yes | Yes | No | Yes | Yes | No | Yes | Yes |  |
| 12. Niyonsenga G. et al, 2021 ([52](file:///C:\Users\user\Desktop\CC%20Review-sept%203,%202022_RKinputs%20-%20(Autosaved).docx#_ENREF_94)) | Yes | Yes | Yes | Yes | No | Yes | Yes | No | Yes | Yes |  |
| 13. Treat JOR. Et al, 2017 ([76](file:///C:\Users\user\Desktop\CC%20Review-sept%203,%202022_RKinputs%20-%20(Autosaved).docx#_ENREF_94)) | Yes | Yes | Yes | Yes | Yes | Yes | Yes | Yes | Yes | No |  |
| 14. Akua E. et al, 2018 ([59](file:///C:\Users\user\Desktop\CC%20Review-sept%203,%202022_RKinputs%20-%20(Autosaved).docx#_ENREF_94)) | Yes | Yes | Yes | Yes | Yes | Yes | Yes | Yes | Yes | Yes |  |
| 15. Cunningham MS. et al, 2014 ([60](file:///C:\Users\user\Desktop\CC%20Review-sept%203,%202022_RKinputs%20-%20(Autosaved).docx#_ENREF_94)) | Yes | Yes | Yes | Yes | Yes | Yes | Yes | Yes | Yes | Yes |  |
| 16. Isabirye A. et al, 2020 ([61](file:///C:\Users\user\Desktop\CC%20Review-sept%203,%202022_RKinputs%20-%20(Autosaved).docx#_ENREF_94)) | Yes | Yes | Yes | Yes | Yes | Yes | Yes | Yes | Yes | Yes |  |
| 17. Wollancho W. et al, 2020 ([73](file:///C:\Users\user\Desktop\CC%20Review-sept%203,%202022_RKinputs%20-%20(Autosaved).docx#_ENREF_94)) | Yes | Yes | Yes | Yes | Yes | Yes | Yes | Yes | Yes | Yes |  |
| 18. Hauwa I. et al, 2021 ([34](file:///C:\Users\user\Desktop\CC%20Review-sept%203,%202022_RKinputs%20-%20(Autosaved).docx#_ENREF_94)) | Yes | Yes | Yes | Yes | No | Yes | Yes | No | Yes | Yes |  |
| 19. Tsegay A, et al, 2019 ([62](file:///C:\Users\user\Desktop\CC%20Review-sept%203,%202022_RKinputs%20-%20(Autosaved).docx#_ENREF_94)) | Yes | Yes | Yes | Yes | Yes | Yes | Yes | Yes | Yes | Yes |  |
| 20. Innocentia Ebu N. et al, 2014 ([67](file:///C:\Users\user\Desktop\CC%20Review-sept%203,%202022_RKinputs%20-%20(Autosaved).docx#_ENREF_94)) | Yes | Yes | Yes | Yes | Yes | Yes | Yes | No | Yes | Yes |  |
| 21. Dulla D. et al, 2017 ([87](file:///C:\Users\user\Desktop\CC%20Review-sept%203,%202022_RKinputs%20-%20(Autosaved).docx#_ENREF_94)) | Yes | Yes | Yes | Yes | Yes | Yes | Yes | Yes | Yes | Yes |  |
| 22. Alemnew W. et al, 2022 ([74](file:///C:\Users\user\Desktop\CC%20Review-sept%203,%202022_RKinputs%20-%20(Autosaved).docx#_ENREF_94)) | Yes | Yes | Yes | Yes | Yes | Yes | Yes | Yes | Yes | Yes |  |
| 23. Ilevbare OE. et al, 2022 ([68](file:///C:\Users\user\Desktop\CC%20Review-sept%203,%202022_RKinputs%20-%20(Autosaved).docx#_ENREF_94)) | Yes | Yes | Yes | Yes | Yes | Yes | Yes | Yes | Yes | Yes |  |
| 24. Osingada CP et al, 2015 ([77](file:///C:\Users\user\Desktop\CC%20Review-sept%203,%202022_RKinputs%20-%20(Autosaved).docx#_ENREF_94)) | Yes | Yes | Yes | Yes | Yes | Yes | Yes | Yes | Yes | Yes |  |
| 25. Okunowo A., 2020 ([35](file:///C:\Users\user\Desktop\CC%20Review-sept%203,%202022_RKinputs%20-%20(Autosaved).docx#_ENREF_94)) | Yes | Yes | Yes | Yes | Yes | Yes | Yes | Yes | Yes | Yes |  |
| \| 26. Boluwatito CO., 2013 \| \| --- \|   (63) | Yes | Yes | Yes | No | Yes | Yes | Yes | No | Yes | Yes |  |
| 27. Boni SP., 2018 ([36](file:///C:\Users\user\Desktop\CC%20Review-sept%203,%202022_RKinputs%20-%20(Autosaved).docx#_ENREF_94)) | Yes | Yes | Yes | Yes | Yes | Yes | Yes | Yes | Yes | Yes |  |
| 28. Bante SA.,  2019 ([75](file:///C:\Users\user\Desktop\CC%20Review-sept%203,%202022_RKinputs%20-%20(Autosaved).docx#_ENREF_94)) | Yes | Yes | Yes | Yes | Yes | Yes | Yes | Yes | Yes | Yes |  |
| 29. Okyere J, 2021 ([83](file:///C:\Users\user\Desktop\CC%20Review-sept%203,%202022_RKinputs%20-%20(Autosaved).docx#_ENREF_94)) | Yes | Yes | Yes | Yes | Yes | Yes | Yes | Yes | Yes | Yes |  |
| 30 Akokuwebe ME, 2021 ([81](file:///C:\Users\user\Desktop\CC%20Review-sept%203,%202022_RKinputs%20-%20(Autosaved).docx#_ENREF_94)) | Yes | Yes | Yes | Yes | Yes | Yes | Yes | Yes | Yes | Yes |  |
| 31. Assoumou SZ,2015 ([70](file:///C:\Users\user\Desktop\CC%20Review-sept%203,%202022_RKinputs%20-%20(Autosaved).docx#_ENREF_94)) | Yes | Yes | Yes | No | Yes | Yes | Yes | No | Yes | Yes |  |
| 32. Tiruneh FN. Et al, 2017 ([84](file:///C:\Users\user\Desktop\CC%20Review-sept%203,%202022_RKinputs%20-%20(Autosaved).docx#_ENREF_94)) | Yes | Yes | Yes | Yes | Yes | Yes | Yes | Yes | Yes | Yes |  |
| 33. Ebu NI., 2018 ([69](file:///C:\Users\user\Desktop\CC%20Review-sept%203,%202022_RKinputs%20-%20(Autosaved).docx#_ENREF_94)) | Yes | Yes | Yes | Yes | Yes | Yes | Yes | Yes | Yes | Yes |  |
| 34. Calys-Tagoe BNL., 2020 ([82](file:///C:\Users\user\Desktop\CC%20Review-sept%203,%202022_RKinputs%20-%20(Autosaved).docx#_ENREF_94)) | Yes | Yes | Yes | Yes | Yes | Yes | Yes | Yes | Yes | Yes |  |
| 35. Wolde-tsadik AB., 2020 ([37](file:///C:\Users\user\Desktop\CC%20Review-sept%203,%202022_RKinputs%20-%20(Autosaved).docx#_ENREF_94)) | Yes | Yes | Yes | Yes | Yes | Yes | Yes | Yes | Yes | Yes |  |
| 36.Obol, 2021 ([86](file:///C:\Users\user\Desktop\CC%20Review-sept%203,%202022_RKinputs%20-%20(Autosaved).docx#_ENREF_94)) | Yes | Yes | Yes | Yes | Yes | Yes | Yes | Yes | Yes | Yes |  |
| 38.Azene, 2021 ([64](file:///C:\Users\user\Desktop\CC%20Review-sept%203,%202022_RKinputs%20-%20(Autosaved).docx#_ENREF_94)) | Yes | Yes | Yes | Yes | Yes | Yes | Yes | Yes | Yes | Yes |  |
| 39.Vhuromu, 2018 ([71](file:///C:\Users\user\Desktop\CC%20Review-sept%203,%202022_RKinputs%20-%20(Autosaved).docx#_ENREF_94)) | Yes | No | Not clear | Yes | Yes | Yes | No | Yes | Yes | Yes |  |
| 40. Gebru Z, 2016 ([78](file:///C:\Users\user\Desktop\CC%20Review-sept%203,%202022_RKinputs%20-%20(Autosaved).docx#_ENREF_94)) | Yes | Yes | Yes | Yes | Yes | Yes | Yes | Yes | Yes | Yes |  |
| 41. Weng, 2020 ([79](file:///C:\Users\user\Desktop\CC%20Review-sept%203,%202022_RKinputs%20-%20(Autosaved).docx#_ENREF_94)) | Yes | Yes | Yes | Yes | Yes | Yes | Yes | Yes | Yes | Yes |  |

1. Additional material: Quality appraisal of the included qualitative studies on cervical cancer screening program implementation.

| **Risk of bias items** | **Lee, 2020** ([41](file:///C:\Users\user\Desktop\CC%20Review-sept%203,%202022_RKinputs%20-%20(Autosaved).docx#_ENREF_94)) | **Adedimeji, 2021** ([42](file:///C:\Users\user\Desktop\CC%20Review-sept%203,%202022_RKinputs%20-%20(Autosaved).docx#_ENREF_94)) | **Adewumi, 2021** ([43](file:///C:\Users\user\Desktop\CC%20Review-sept%203,%202022_RKinputs%20-%20(Autosaved).docx#_ENREF_94)) | **Ebu, 2018** ([44](file:///C:\Users\user\Desktop\CC%20Review-sept%203,%202022_RKinputs%20-%20(Autosaved).docx#_ENREF_94)) | **Osei, 2021** ([72](file:///C:\Users\user\Desktop\CC%20Review-sept%203,%202022_RKinputs%20-%20(Autosaved).docx#_ENREF_94)) | **Mukuku, 2020** ([45](file:///C:\Users\user\Desktop\CC%20Review-sept%203,%202022_RKinputs%20-%20(Autosaved).docx#_ENREF_94)) | **Mpata, 2021** ([85](file:///C:\Users\user\Desktop\CC%20Review-sept%203,%202022_RKinputs%20-%20(Autosaved).docx#_ENREF_94)) | **Teng, 2014** ([46](file:///C:\Users\user\Desktop\CC%20Review-sept%203,%202022_RKinputs%20-%20(Autosaved).docx#_ENREF_94)) | **Linde, 2019** ([65](file:///C:\Users\user\Desktop\CC%20Review-sept%203,%202022_RKinputs%20-%20(Autosaved).docx#_ENREF_94)) | **Roux, 2021** ([55](file:///C:\Users\user\Desktop\CC%20Review-sept%203,%202022_RKinputs%20-%20(Autosaved).docx#_ENREF_94)) |
| --- | --- | --- | --- | --- | --- | --- | --- | --- | --- | --- |
| 1. Was there a clear statement of the aims of the research? | Yes | Yes | Yes | Yes | Yes | No | Yes | Yes | Yes | Yes |
| 1. Is a qualitative methodology appropriate? | Yes | Yes | Yes | Yes | Yes | Yes | Yes | Yes | Yes | Yes |
| 1. Was the research design appropriate to address the aims of the research? | Yes | Yes | Yes | Yes | Yes | Unclear | Yes | Yes | Yes | Unclear |
| 1. Was the recruitment strategy appropriate to the aims of the research? | Yes | Yes | Yes | Yes | Yes | Yes | Yes | Yes | Yes | Yes |
| 1. Was the data collected in a way that addressed the research issue? | Yes | Yes | Yes | Yes | Yes | Yes | Yes | Yes | Yes | Yes |
| 1. Has the relationship between researcher and participants been adequately considered? | Yes | Yes | Yes | Yes | Yes | Yes | Yes | Yes | Yes | Yes |
| 1. Have ethical issues been taken into consideration? | Yes | Yes | Yes | Yes | Yes | Yes | Yes | Yes | Yes | Yes |
| 1. Was the data analysis sufficiently rigorous? | Yes | Yes | Yes | Yes | Yes | Yes | Yes | Yes | Yes | Yes |
| 1. Is there a clear statement of findings? | Yes | Yes | Yes | Yes | Yes | Yes | Yes | Yes | Yes | Yes |
| 1. How valuable is the research? | Yes | Yes | Yes | Yes | Yes | Yes | Yes | Yes | Yes | Yes |
| Percentage score (criteria met) |  |  |  |  |  |  |  |  |  |  |
| Overall quality |  |  |  |  |  |  |  |  |  |  |

**Sources: CASP +** EPHPP

Additional material: Quality appraisal of the included qualitative studies on cervical cancer screening program implementation. (**CONTNUE).**

| **Risk of bias items** | **Matenge, 2018** ([47](file:///C:\Users\user\Desktop\CC%20Review-sept%203,%202022_RKinputs%20-%20(Autosaved).docx#_ENREF_94)) | **Major, 2018** ([48](file:///C:\Users\user\Desktop\CC%20Review-sept%203,%202022_RKinputs%20-%20(Autosaved).docx#_ENREF_94)) | **McCree, 2015** ([90](file:///C:\Users\user\Desktop\CC%20Review-sept%203,%202022_RKinputs%20-%20(Autosaved).docx#_ENREF_94)) | **Bukirwa et al., 2015** ([49](file:///C:\Users\user\Desktop\CC%20Review-sept%203,%202022_RKinputs%20-%20(Autosaved).docx#_ENREF_94)) | **Mensah, 2020** ([50](file:///C:\Users\user\Desktop\CC%20Review-sept%203,%202022_RKinputs%20-%20(Autosaved).docx#_ENREF_94)) | **Lott et al, 2021** ([88](file:///C:\Users\user\Desktop\CC%20Review-sept%203,%202022_RKinputs%20-%20(Autosaved).docx#_ENREF_94)) | **Datchoua Moukam et al, 2021** ([53](file:///C:\Users\user\Desktop\CC%20Review-sept%203,%202022_RKinputs%20-%20(Autosaved).docx#_ENREF_94)) |
| --- | --- | --- | --- | --- | --- | --- | --- |
| 1. Was there a clear statement of the aims of the research? | Yes | Yes | Yes | Yes | Yes | No | Yes |
| 1. Is a qualitative methodology appropriate? | Yes | Yes | Yes | Yes | Yes | Yes | Yes |
| 1. Was the research design appropriate to address the aims of the research? | Yes | Unclear | Unclear | Unclear | Yes |  | Yes |
| 1. Was the recruitment strategy appropriate to the aims of the research? | Yes | Yes | Yes | Yes | Yes | Yes | Yes |
| 1. Was the data collected in a way that addressed the research issue? | Yes | Yes | Yes | Yes | Yes | Yes | Yes |
| 1. Has the relationship between researcher and participants been adequately considered? | Yes | Yes | Yes | Yes | Yes | Yes | Yes |
| 1. Have ethical issues been taken into consideration? | Yes | Yes | Yes | Yes | Yes | Yes | Yes |
| 1. Was the data analysis sufficiently rigorous? | Yes | Yes | Yes | Yes | Yes | Yes | Yes |
| 1. Is there a clear statement of findings? | Yes | Yes | Yes | Yes | Yes | Yes | Yes |
| 1. How valuable is the research? | Yes | Yes | Yes | Yes | Yes | Yes | Yes |
| Percentage score (criteria met) |  |  |  |  |  |  |  |
| Overall quality |  |  |  |  |  |  |  |

1. Additional material: Quality appraisal of the included **mixed-methods studies (MMAT)** on cervical cancer screening program implementation.

| **Risk of bias items** | Ducray, 2021 ([38](file:///C:\Users\user\Desktop\CC%20Review-sept%203,%202022_RKinputs%20-%20(Autosaved).docx#_ENREF_94)) | Getachew et al, 2019 (39) | Shiferaw etal, 2018 ([40](file:///C:\Users\user\Desktop\CC%20Review-sept%203,%202022_RKinputs%20-%20(Autosaved).docx#_ENREF_94)) |
| --- | --- | --- | --- |
| 1. Is there an adequate rationale for using a mixed methods design to address the research question? | Yes | Yes | Yes |
| 1. Are the different components of the study effectively integrated to answer the research question? | Yes | Yes | Yes |
| 1. Are the outputs of the integration of qualitative and quantitative components adequately interpreted? | Yes | Yes | Yes |
| 1. Are divergences and inconsistencies between quantitative and qualitative results adequately addressed? | Yes | Yes | Yes |
| 1. Do the different components of the study adhere to the quality criteria of each tradition of the methods involved? | Yes | Yes | Yes |
| Percentage score (criteria met) | 100 | 100 | 100 |
| Overall quality |  |  |  |

**Supporting information 3: Characteristics and key findings of the included studies**

1. **Individual (intrapersonal) factors**

| **Individual (intrapersonal) level factors** | **Factors examined** | **Key results** | | | **Country/region** |
| --- | --- | --- | --- | --- | --- |
|  |  | **Barriers identified** | **Facilitators identified** | **Recommendation** |  |
| **Awareness and understanding of cervical cancer and its screening** | Knowledge ([31,32,33,34,37,38,39,40,41, 42,43,44,45,46,48,49,50,51,5354, 55,56,57,61,63,64,65,66,72,](file:///C:\Users\user\Desktop\CC%20Review-sept%203,%202022_RKinputs%20-%20(Autosaved).docx#_ENREF_94)88)  ever received cancer information ([62](file:///C:\Users\user\Desktop\CC%20Review-sept%203,%202022_RKinputs%20-%20(Autosaved).docx#_ENREF_94)) | Low awareness & knowledge about CC & its screening ([31,32,33,34,35,36,37,38,39,40,42,49,50](file:///C:\Users\user\Desktop\CC%20Review-sept%203,%202022_RKinputs%20-%20(Autosaved).docx#_ENREF_94)) unaware of where to be screened ([54](file:///C:\Users\user\Desktop\CC%20Review-sept%203,%202022_RKinputs%20-%20(Autosaved).docx#_ENREF_94)), poor knowledge on  availability of screening services ([52](file:///C:\Users\user\Desktop\CC%20Review-sept%203,%202022_RKinputs%20-%20(Autosaved).docx#_ENREF_94)), Poor knowledge and misunderstanding ([41](file:///C:\Users\user\Desktop\CC%20Review-sept%203,%202022_RKinputs%20-%20(Autosaved).docx#_ENREF_94)),  Confusion between human papillomavirus (HPV) & HIV ([46](file:///C:\Users\user\Desktop\CC%20Review-sept%203,%202022_RKinputs%20-%20(Autosaved).docx#_ENREF_94)), lack of health literacy (53,[55](file:///C:\Users\user\Desktop\CC%20Review-sept%203,%202022_RKinputs%20-%20(Autosaved).docx#_ENREF_94)), and misconceptions ([88](file:///C:\Users\user\Desktop\CC%20Review-sept%203,%202022_RKinputs%20-%20(Autosaved).docx#_ENREF_94)) | Being knowledgeable ([56,57,58,59,60,61,62,63,65](file:///C:\Users\user\Desktop\CC%20Review-sept%203,%202022_RKinputs%20-%20(Autosaved).docx#_ENREF_94)), Knowing methods of CC prevention ([66](file:///C:\Users\user\Desktop\CC%20Review-sept%203,%202022_RKinputs%20-%20(Autosaved).docx#_ENREF_94)), knowing the availability and place of cervical cancer screening service ([51,37](file:///C:\Users\user\Desktop\CC%20Review-sept%203,%202022_RKinputs%20-%20(Autosaved).docx#_ENREF_94)), knowledge on the consequence of advanced cervical cancer ([51](file:///C:\Users\user\Desktop\CC%20Review-sept%203,%202022_RKinputs%20-%20(Autosaved).docx#_ENREF_94)), awareness about cervical cancer and VIA screening ([64](file:///C:\Users\user\Desktop\CC%20Review-sept%203,%202022_RKinputs%20-%20(Autosaved).docx#_ENREF_94)) | Awareness creation ([31,33,36,57,58,59,61,62,66](file:///C:\Users\user\Desktop\CC%20Review-sept%203,%202022_RKinputs%20-%20(Autosaved).docx#_ENREF_94)) health workers should go out  into the community and educate women ([85](file:///C:\Users\user\Desktop\CC%20Review-sept%203,%202022_RKinputs%20-%20(Autosaved).docx#_ENREF_94)), enhancing health literacy by strengthening community health activities ([53](file:///C:\Users\user\Desktop\CC%20Review-sept%203,%202022_RKinputs%20-%20(Autosaved).docx#_ENREF_94))  regular house visits,  awareness campaigns, mass media, and sensitization  by scheme staff, scheme members and local churches | Nigeria ([31,34,63](file:///C:\Users\user\Desktop\CC%20Review-sept%203,%202022_RKinputs%20-%20(Autosaved).docx#_ENREF_94)), Rwanda (52,58), Tanzania ([54,65](file:///C:\Users\user\Desktop\CC%20Review-sept%203,%202022_RKinputs%20-%20(Autosaved).docx#_ENREF_94)), Ethiopia (33,37,39,40.64,51,[57,62,66,88](file:///C:\Users\user\Desktop\CC%20Review-sept%203,%202022_RKinputs%20-%20(Autosaved).docx#_ENREF_94)) Kenya (43,[59](file:///C:\Users\user\Desktop\CC%20Review-sept%203,%202022_RKinputs%20-%20(Autosaved).docx#_ENREF_94)) , Uganda (46,49,[51](file:///C:\Users\user\Desktop\CC%20Review-sept%203,%202022_RKinputs%20-%20(Autosaved).docx#_ENREF_94)), Côte d’Ivoire ([36,50](file:///C:\Users\user\Desktop\CC%20Review-sept%203,%202022_RKinputs%20-%20(Autosaved).docx#_ENREF_94)), Malawi ([41](file:///C:\Users\user\Desktop\CC%20Review-sept%203,%202022_RKinputs%20-%20(Autosaved).docx#_ENREF_94)), South Africa ([38](file:///C:\Users\user\Desktop\CC%20Review-sept%203,%202022_RKinputs%20-%20(Autosaved).docx#_ENREF_94)), Cameroon (42,53,55,), Ghana ([44,72](file:///C:\Users\user\Desktop\CC%20Review-sept%203,%202022_RKinputs%20-%20(Autosaved).docx#_ENREF_94)), DRC (45,) Botswana ([47,48](file:///C:\Users\user\Desktop\CC%20Review-sept%203,%202022_RKinputs%20-%20(Autosaved).docx#_ENREF_94)) |
| **Accessibility of health information on CC & screening** |  | Lack of access to information on CC & screening (10, 12, 25,46,50,61), | Had got CC information (35), |  | Ethiopia (10,35), Cameroon(46) |
| **Risk perceptions and healthcare related behaviors** | Perceived susceptibility (1, 6, 18, 20, 23, 26, 33, 38, 40, 46) | Unlikely chance of having cancer (20), low perception of risk (18, 46), poor health seeking behaviors (46), perceiving as not being susceptible (48) | Good/positive perception status about CC increased CC screening uptake (1, 6), perceived risk or susceptible or fear of getting cervical cancer (26, 35, 38, 48), perceived seriousness & severity of CC (23, 33, 40), cues about CC increased the intention of screening (33). | Improving perception through behavioral change & communication (1,6) | Nigeria (1, 18,23,26), Ethiopia (6,38,40), Ghana (22,33), Cameroon (46), Ghana (48) |
|  | Perceived benefit (33, 53) | Low perceived importance to have screening (55) | Perceived benefits increased intention of screening (33),  Perceived benefits for attending a patient initiated  screening include treatment of gynecological  symptoms and prevention of disease (53) |  | Ghana (33)  Tanzania (53), Botswana (55) |
|  | Perceived fear (10, 11,23,27,31,35,39,42,44,47, 50,52,53,55,56,58,59) | Fear of screening test results (10, 23, 27, 31,35,42,44,50,59), fear of pain of procedure (11,39,42,44,47,55), worry about results and stigma (52), emotional costs in the form of fear of the disease (53,56), fear of the gynecological examination & procedure (4,10, 53), fear of the unknown (56), myths and misconceptions (fear of removing womb) (58) |  |  | Ethiopia (10,35,44), Tanzania (11,53),  Nigeria (23), Cote d’Ivoires (27,59), Gabon (31), South Africa (39,42), Kenya (4), DRC (50), Uganda (52,58), Botswana (55,56), |
|  | Embarrassment (4, 39,47,49,52,58) | Feeling shy (4,39,47,49), Location of self-collection or privacy (52), Sexual organs are private  (56), fear of invasion into their privacy (58) |  |  | Ethiopia (10,), South Africa (39), Kenya (47), Ghana (49), Uganda (52,58), Botswana (56) |
|  | Attitude of women towards screening (17, 28, 49) | Carelessness or negligence (27), neglect (31), deeming it unimportant (31), being virgin hence not legible for screening (49), | Direct and indirect attitude to intent for screening (17), direct attitude (22), Positive attitude (23, 28) | Conduct Social and Behavioral Change communication (17) | Ethiopia (17, 22,28), Nigeria (23), Cote d’ Ivories (27), Gabon (31), Ghana (49) |
|  | Level of involvement with healthcare (34) | Moderate and below rate healthcare involvement (34) |  |  |  |
|  | Women sense of behavioral control (17,22) |  | Direct and indirect perceived  behavioral control to intend for screening (17), Direct perceived behavioral control to intend screening (22) | Increase their sense of control (17), behavioral change communication interventions (22), evaluating behavioral control and normative beliefs (22) | Ethiopia (17, 22) |
| **Socio-demographic factors** | Age (3,6, 10, 13, 14, 15, 24,27,28,35,38,40,41,49,52) | 35 years or older had lower odds of utilization of screening (14), | Being older age (3, 15, 38), age  between 21 and 29 years old (6), Being in the age group of 30–39 years (10,13), age greater than 25 years (24), being ≥45 years (27), Participants ages 35–49 (28), being between 24–45 years old (29), age above 35 years (30), between 40–49 years old (35), age of mother greater than 30 years (40), Young age (49) |  | Ethiopia (13,14, 28,35,38,40)), Tanzania (15,41),  Côte d’Ivoire (27),  Cameroon (29)  Ghana (49), Uganda (52) |
|  | Educational status (5,8,13, 19,27,29, 30, 34, 41) |  | women with primary& above education (5,8),  Secondary school (19), college and above level of education (13,18, 27, 29,30), husband education (34) | Increasing women’s formal education (5,8), improving education at the community level (32) | Ethiopia (5,13), Nigeria (18), Côte d’Ivoire (27), Cameroon (29), South Africa (30), Gabon (32), Ghana (34), Tanzania (41) |
|  | Economic condition (7,13, 16, 19, 26, 29,31,32,35,40,41,42,44, 50,54) | Lack of financial resources (31,32,42,48), low monthly income (35), financial constraints (44,54), lack of money (50) | Earning RWF ≥ 63,751 (7), Annual household income of more than 30,000 ETB (13), rich women (16,29), perceived income of the household rich (19, 26), higher household wealth index (32), average monthly income of greater than 1170 ETB (40) | Empowering women, and providing employment opportunities for women (32) | Rwanda (7,13), Uganda (16), Ethiopia (19,35,40, 44), Nigeria (26), Cameroon (29,54), Gabon (31), Kenya (32), Tanzania (41), South Africa (42), Ghana (48), DRC (50) |
|  | Occupation (3,30,32) |  | Being government employed (3, 30), were employed (32) |  | South Africa (30), Kenya (32) |
|  | Residence (12,13, 29,35) | Living in a rural area (12) | Dwelling in urban area (13, 29, 35) |  | Ethiopia (13,35), Cameroon (29), Rwanda (12) |
|  | Marital Status (29, 30,34,41) | Married & widowed (34) | Being married (29), Divorced (30) |  | Cameroon (29), South Africa (30), Ghana (34), Tanzania (41) |
|  | Ethnicity (30) |  | White population group (30), Ewe & Guan (34) |  | South Africa (30), |
| **Personal pre- disposition (temperament)** | Perceived current health status (11,39,40,43,44,49,51) | Having no sign & symptoms (11,43), being/fleeing healthy (40,44,49) | Being ill (39), having signs and symptoms of cervical cancer (51), Cell differentiation 4 (CD4) count of less than or equal to 200 cell/ mm3 (5), diagnosed as HIV positive ten years back or more (5), being HIV positive (51), role of HIV status in cancer (59), unknown family tumor (41) | Strengthening healthcare workers' capacity to promote cervical cancer awareness (11); integrate the screening service to the routine care and treatment and provide counseling services (5) | Tanzania (11), South Africa (39), Ethiopia (5,40,43, 44,49), Zimbabwe (51), Cote D Ivories (59) |
|  | History of illness (8, 26, 28,35,38, 41) | Do not get sick previously (10) | Family history of CC (26, 28), history of sexually transmitted infections (28,38), history of CC illness (35), history of schistosomiasis (41), history of family genetic disorder (41) | STI clinic should be linked to the cervical cancer |  |
|  | History of practicing prevention (early detection test) (22,29) and taking therapy |  | Having previous screening experience (22), Ever undergone HIV test (29), |  | Cameroon (29), Ethiopia (22) |
|  | Contraceptive use (24) | Not using hormonal contraceptives (24), | Using of family planning (3) | Promoting & linking CC in the maternal services | Uganda (24) |
|  | History of visiting health facilities (28, 32) |  | Visited health institution once or more in a year or above (28), visit a health facility in 12 month (32), visiting the gynecology departments (3), attending private healthcare facilities (3) | Service linkage among departments | Ethiopia (28), Kenya (32) |
| Lifestyle factors | Bad lifestyle behaviors | Substance abuse (55) |  |  |  |
|  |  |  | Women who have multiple sexual partners (8) |  | Ethiopia (8) |

1. **Interpersonal factors**

| **Interpersonal** | **Factors examined** | **Key Results** | | | **Country/ region** |
| --- | --- | --- | --- | --- | --- |
|  |  | **Barriers identified** | **Facilitators identified** | **Recommendation** |  |
| Peer influence | Peer persuasion (4, 47,51) |  | Getting advice from friends and relatives (25), Peer pressure (47), Presence of peers or relatives to model preventive behavior (47), | Peer health education (51) | Ethiopia (10), Nigeria (25), Kenya (47), Zimbabwe (51) |
|  | Couple influence (39) | Partners refusal (disapproval) (4, 10), Husbands did not support Pap smear screening (39) | Support and encouragement from the spouse (51) | Husband education | South Africa (39) |
|  | Peer attitude (17) |  | Direct and indirect subjective norm (17), direct subjective norm (22) | Improve the  attitude of influential peers & peoples (17) | Ethiopia (17, 22) |
| Household dynamic | Household size (5) & parity (40,41) | Parity of more than 7 births (41) | Had children (5,51), parity of more than 5 children (40), |  | Zimbabwe (51), Ethiopia (40), Tanzania (41) |
| Network & relationship | Network with peer |  | Utilization of peer  networks to model screening behavior (47), |  | Kenya (47) |
|  | Relation with relevant others (52) | Relationship with health worker (52) |  |  | Uganda (52) |

1. **Organizational (providers) factors**

| **Organizational (provider)** | **Factors examined** | **Key Results** | | |  |
| --- | --- | --- | --- | --- | --- |
|  |  | **Barriers identified** | **Facilitators identified** | **Recommendation** | **Country/region** |
| **Providers or organizational level of awareness and understanding on cervical cancer and its screening** | Knowledge, communication, and counseling skill of provider (3,10,58,60,61) | Lack of awareness campaign on screening (12), Low provider-awareness of cervical cancer and screening (60), Providers lack of knowledge (47), Inadequate provision of health education about cervical cancer (58), Poor patient-centered communication skills (55) | Good professionals’ ability of counseling of clients (3), Facilitation of discussion forum with clients (10) | Working on provider’s education  (3,10,12), providing training for HCPs and community healthcare workers to improve patient-provider-communication (61), Strengthen discussion form & regular health education in the facilities (3,12,60) | Rwanda (12), Uganda (58), Ethiopia (60), Cameroon (61) |
|  | Source of information (16,38) |  | Receiving information from healthcare providers (16,38), Receiving community health education from health extension workers (38), |  | Ethiopia (38), Uganda (16), Botswana (55) |
| **Risk perceptions and healthcare related behaviors of providers** | Attitudes of healthcare providers | Bad attitude about CC (4,55,60), negative attitudes of providers toward clients (12), low work commitments (55) |  | Attitudes of healthcare providers | Kenya (4), Rwanda (12) Ethiopia (60) |
|  | Provider recom mendations (18, 24, 28,42, 44,53,59) | Screening not recommended by a health worker (18,44), Never received encouragement (24), Were never told to have a Pap smear (42), | Referral & linkage by provider (20),  Recommendation by doctors/nurses (25, 28), Provider-initiated follow-up screening (53), | Universal recommendation of the  HPV screening test should be routinized & integrated to other services | Nigeria (18,18), Ghana (20), Uganda (24), Ethiopia (28,44), South Africa (42), Tanzania (53), |
| Socio-demographic of providers | The role of gender on CCS (4,23,52,60) | Male provider (4,23) | Female provider (24,60) | increasing the number of female providers | Nigeria (23), Uganda (24,52), Ethiopia (60) |
|  | Training status (36,56) | Lack of suitable training for all service providers (56) | Had been trained to screen for cervical cancer (36) | Training health workers by incorporating in pre-service healthcare training curricula (36) | Uganda (36), Botswana (56) |
|  | Type of profession (21) | Being physician (21) |  | Increasing the number of nurse-midwife providers | Ethiopia (21) |
| Working environment and level of healthcare facility | Type of working department (21) | working in cervical cancer screening center (21) |  | Providers education | Ethiopia (21) |
|  | Level of working setups (36) |  | Working in a staff member from Health center level III (36) |  | Uganda (36) |
|  | Organizational support (36) |  | Working in health centers that has organization support for cervical cancer screening service (36) |  | Uganda (36) |

1. **Community (Societal) level factors**

| **Community (societal)** | **Factors examined** | **Key Results** | | | **Country/region** |
| --- | --- | --- | --- | --- | --- |
|  |  | **Barriers identified** | **Facilitators identified** | **Recommendation** |  |
| Sociocultural norm & beliefs | Beliefs and misconceptions on the cause of cervical cancer | Beliefs on no gynecological sign as absence of disease and no need of screening (45), socio-cultural norms (46,57), wrong norms and beliefs about CC (56) |  | Education about cervical cancer screening | Malawi (45), Cameroon (46), Botswana (56), Tanzania (57) |
|  | HIV and sexuality and gender-based stigma (12,46) | Social stigmatization (12), HIV-related stigma when screening is integrated into HIV care (46), cervical cancer associated with women’s sexuality and reproductive organs contribute to stigma (46) |  | Community education and stigma reduction around cervical  cancer (46) | Rwanda (12), Cameroon (46), |
|  | Religion and cultural values (20,23), | Religion and cultural values (20, 23), Some Christian churches did not approve women to go for cervical cancer screening, (56) |  | Drafting cultural friendly cervical cancer screening policies & strategy | Ghana (20), Nigeria (23), Botswana (56), |
| Community and social networks | Social network |  | Social network was an interface of social learning of screening behavior (46,47) | Utilize existing social networks to  expand literacy of CC risk and screening (47) | Cameroon (46) |

1. **Healthcare system & policy level factors**

| **Healthcare system & policy level factors** | **Factors examined** | **Key Results** | | | **Country/region** |
| --- | --- | --- | --- | --- | --- |
|  |  | **Barriers identified** | **Facilitators identified** | **Recommendation** |  |
| Infrastructures, service availability & resource allocation | Infrastructure & service availability (4, 18, 42,43,39) | Lack of clinics & facilities (39,18), Absence of mobile clinics (42), lack of a screening service in their living area (4,43) |  | The number of mobile clinics should be increased (42), provide alternative accessible facilities (39) | Nigeria (18), South Africa (39,42), Ghana (4), Ethiopia (43) |
|  | Resource allocation (logistics) (45,46,47,50,54,55,58,59,60,61) | Shortages of medical supplies and equipment (45,46,47,54,55,60,61), lack of screening and diagnostic tools (50), stock out of  supplies (58), insufficient resources for treatment (59), limited financial resources (57) |  |  | Malawi (45), Cameroon (46,54,61), Ethiopia (60), Kenya (47), DRC (50), Botswana (55), Uganda (58,59), Tanzania (57), |
| Cost of cervical cancer screening and payment arrangement | Cost of care and cost-sharing (4, 15,27,46,51,61) | Cost of the screening procedure (4,51,61), fear of additional costs (27), unable to afford the test or the travel costs (15), excessive cost of cervical cancer screening (46), direct  and indirect economic costs (53) | Free CC screening (2), free treatment for cancer (51), fees removal (59), free payment arrangement for CC screening in the weekend (4) | Scale upping free services (2), Promoting of publicly funded cervical cancer screening programs, Provision of affordable and  quality care (51) | Cote d’ Ivories’ (27,59), Malawi (46), Zimbabwe (51), Tanzania (53), Cameroon (61) |
| Human resources & training | Availability of trained staff | Shortage of human resources (45,57), lack of trained staff (47), no skilled staff (no those who can read the smears) (50,54,55), lack of suitable training for all service providers (56), high turn-over of providers and medical directors (60) | Female health workers available to screen (60) | Increasing the number of trained health workers (57), | Malawi (45), Kenya (47), DRC (50,56), Botswana (56), Tanzania (57), Ethiopia (60) |
| Human resource motivation & incentive package arrangement | Incentive packages |  | Financial incentive packages increased CC screening coverage (2) | Scale up of incentives for provider (2) |  |
| Awareness creation strategy, policy & its accessibility |  | Absence of health education programs (20) | Provision of text message (2), radio campaign (20), enlightenment by the media (25), access to mass campaign information (27), media exposure (32), access to information and having trust in the health delivery system (51) | Scale up of health education on CC (2), suggested that television and radio were  valuable media (51), development of clear and simple educational messages (51), variable support from administration at institutional,  local, regional, and national levels (60) | Ghana (20), Nigeria (25),  Cote d ’Ivories (27), Kenya (32), Zimbabwe (51), Ethiopia (60) |
| Time of service delivery | Time constraints | Long waiting time for screening  (Long queues) (12), waiting time (53,58), unavailability of test results promptly (delaying results) (55,56), & overloaded and busy healthcare clinics (42), Providers reported workload (47) |  |  | Rwanda (12), Tanzania (53), Uganda (58), Botswana (55,56), South Africa (42), Kenya (47) |
| Service delivery system | Sustainability | Inconsistent appointment systems (55), donor‑dependent system (45),  gaps in the health system (57) | Service delivery system (57), building partnerships and leveraging (57) | Improving access to screening and treatment (57), garnering political (57) | Botswana (55), Malawi (45), Tanzania (57) |
|  | Quality of screening services | Level of satisfaction with healthcare | Dissatisfied/ very dissatisfied with screening services accompanied low screening use (34) |  | Ghana (34), |
|  | Perceived quality of screening (24, 27), Novelty of the test (52) | Perceived screening services as poor (24), Fear of bad reception at CC screening facility (27), poor perceived quality of care (54), poor effectiveness of treatment (55) |  |  | Uganda (24), Cote d’ ivories’ (27), Uganda (52), Cameroon (54), Botswana (55) |
| Geographic reach access | Distance to facility | Long distance from healthcare facility (screening facility & distribution of facilities) (12, 15, 20,45,54), distance, public transport issues limit (55), low geographic accessibility of the program (61) |  | Increase accessibility of CC (12) | Ghana (20), Malawi (45), Cameroon (54,61), Botswana (55) |
| Space and infrastructure |  | Inadequate space & no room available to screen (60) |  |  | Ethiopia (60) |
| Social protection policy |  | Lack of health insurance (12) | Having health insurance coverage (15, 30,32) | Increasing universal health insurance coverage and equity (32) | Tanzania (15), South Africa (30), Kenya (32) |
| Policy related factors | Policy | Lack of a national policy (34), lack of national cancer prevention policies and programs (46), there is no policy, framework & structure that support screening (50), poor referral systems (54), Lack of a proper follow-up mechanism (58) | Change at a system (47), Political will (57), decentralizing health services (57) |  | Ghana (34), Cameroon (46,54), Kenya (47), DRC (50), Tanzania (57), Uganda (58) |
|  |  |  | availability of alternatives to the pelvic exam (47) |  | Kenya (47) |
|  |  |  | integration of cervical cancer screening with other services such as HIV screening campaigns (47,51) | integration of cervical cancer screening into HIV (51) | Kenya (47), Zimbabwe (51) |
|  |  | Place of recruitment (52) |  |  | Uganda (52) |
